# Supplementary material for: The sugarcane mitochondrial genome: assembly, phylogenetics and transcriptomics
Source: PeerJ. 2019 Sep 24;7:e7558. doi: 10.7717/peerj.7558 (PMC6764373; doi:10.7717/peerj.7558)
Supplement: Supplemental Information 1 — Full analysis of the potential new CMS2 cytoplasmic male sterility factor in sugarcane, including amplification primers for CMS2 mitochondrial regions, protein alignment comparisons and domain mapping, analysis of domain origins and expression analyses. [file peerj-07-7558-s001.pdf]

Supplemental Document 1: Assembly Graphs for *Saccharum spontaneum* SES234B and *Miscanthus sinensis* cv Andante based on the mitogenome of *Saccharum* hybrid SP80-3280

Figure 1. Assembly graph for the *Saccharum spontaneum* SES234B mapped by blast to the *Saccharum* hybrid SP80-3280 mitogenome chromosome 1.

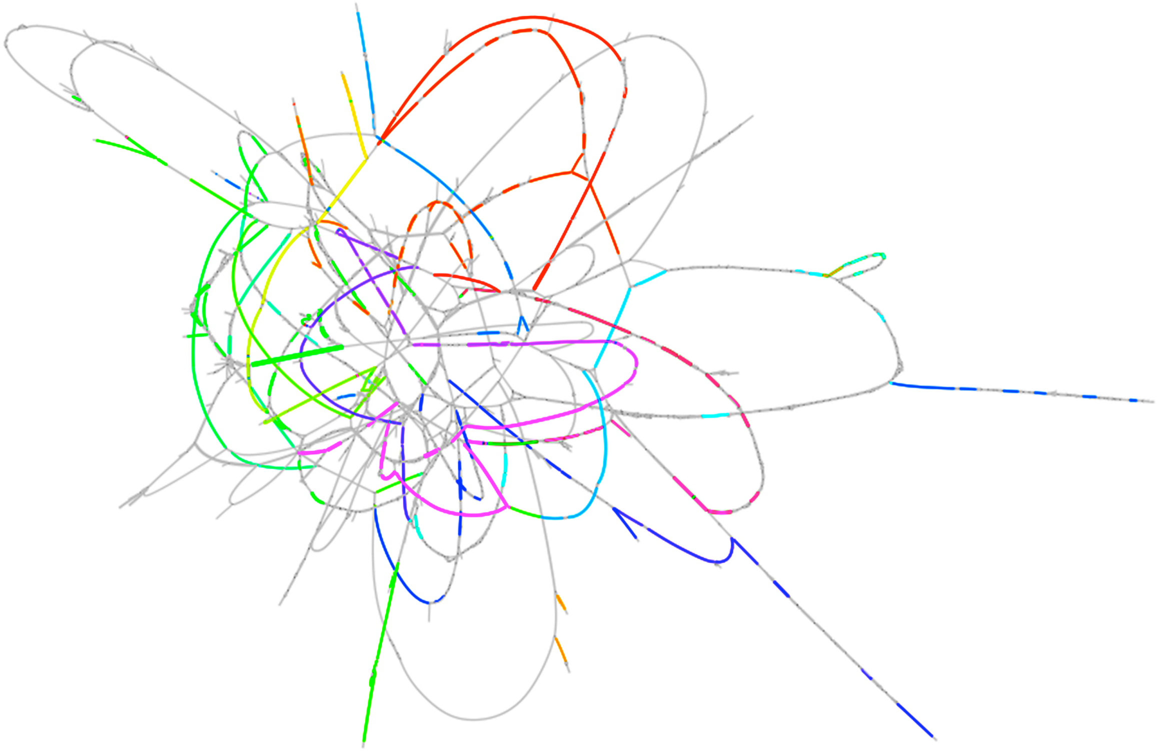

Assembly graph from SPAdes drawn with Bandage for the assembly of the *Saccharum spontaneum* SES234B mitogenome. The *Saccharum* hybrid SP80-3280 mitochondrial chromosome 1 was mapped by BLAST and is shown in colour in the figure. Unmapped contigs are shown in grey. The sugarcane chromosome is both fragmented and has insertions as compared with the *S. spontaneum* assembly.

Figure 2. Assembly graph for the *Saccharum spontaneum* SES234B mapped by blast to the *Saccharum* hybrid SP80-3280 mitogenome chromosome 2.

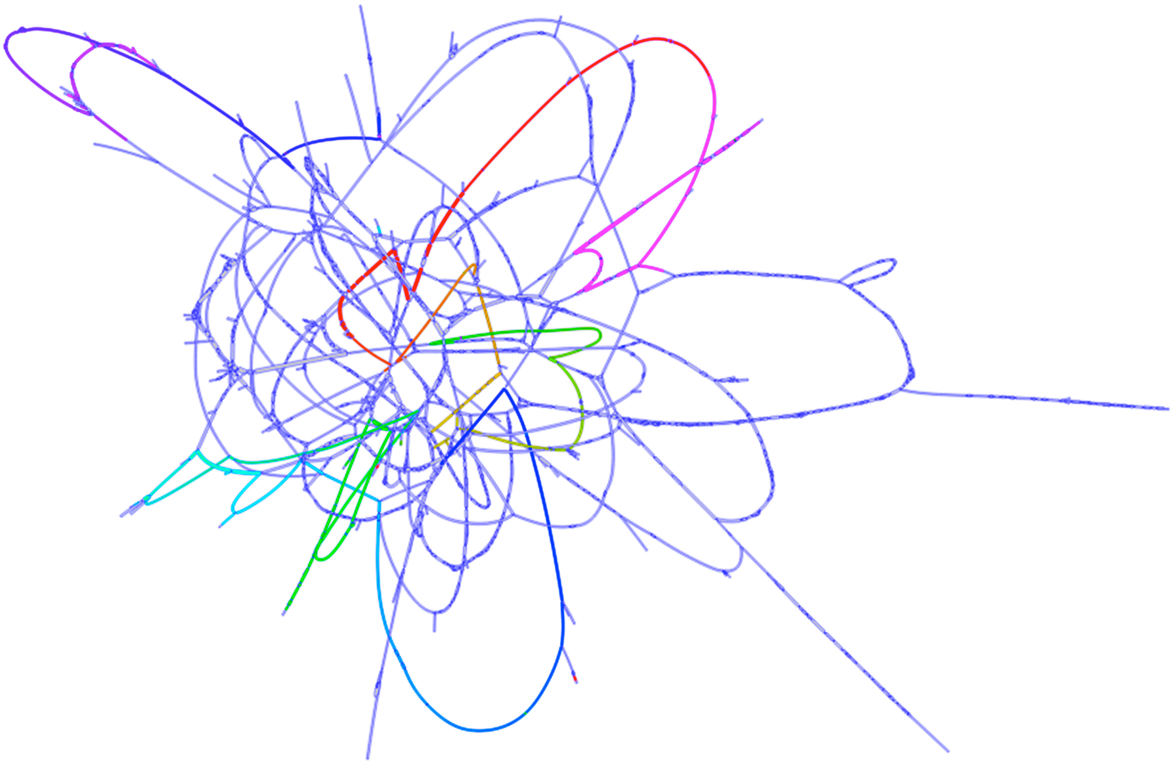

Assembly graph from SPAdes drawn with Bandage for the assembly of the *Saccharum spontaneum* SES234B mitogenome. The *Saccharum* hybrid SP80-3280 mitochondrial chromosome 2 was mapped by BLAST and is shown in colour in the figure. Unmapped contigs are shown in light blue. The sugarcane chromosome is both fragmented and has insertions as compared with the *S. spontaneum* assembly.

Comparisons of the two graphs above (Figure 1 and Figure 2) shows that there are separate contigs corresponding to the sugarcane mitochondrial chromosomes 1 and 2 in the *S. spontaneum* assembly graphs. However, the chromosomes as mapped are not contiguous and there are contigs joining them. Thus the structure of the *S. spontaneum* mitogenome is different from that of the sugarcane mitogenome.

Figure 3. Assembly graph for the *Miscanthus sinensis* cv Andante mitogenome mapped by blast to the *Saccharum* hybrid SP80-3280 mitogenome chromosome 1.

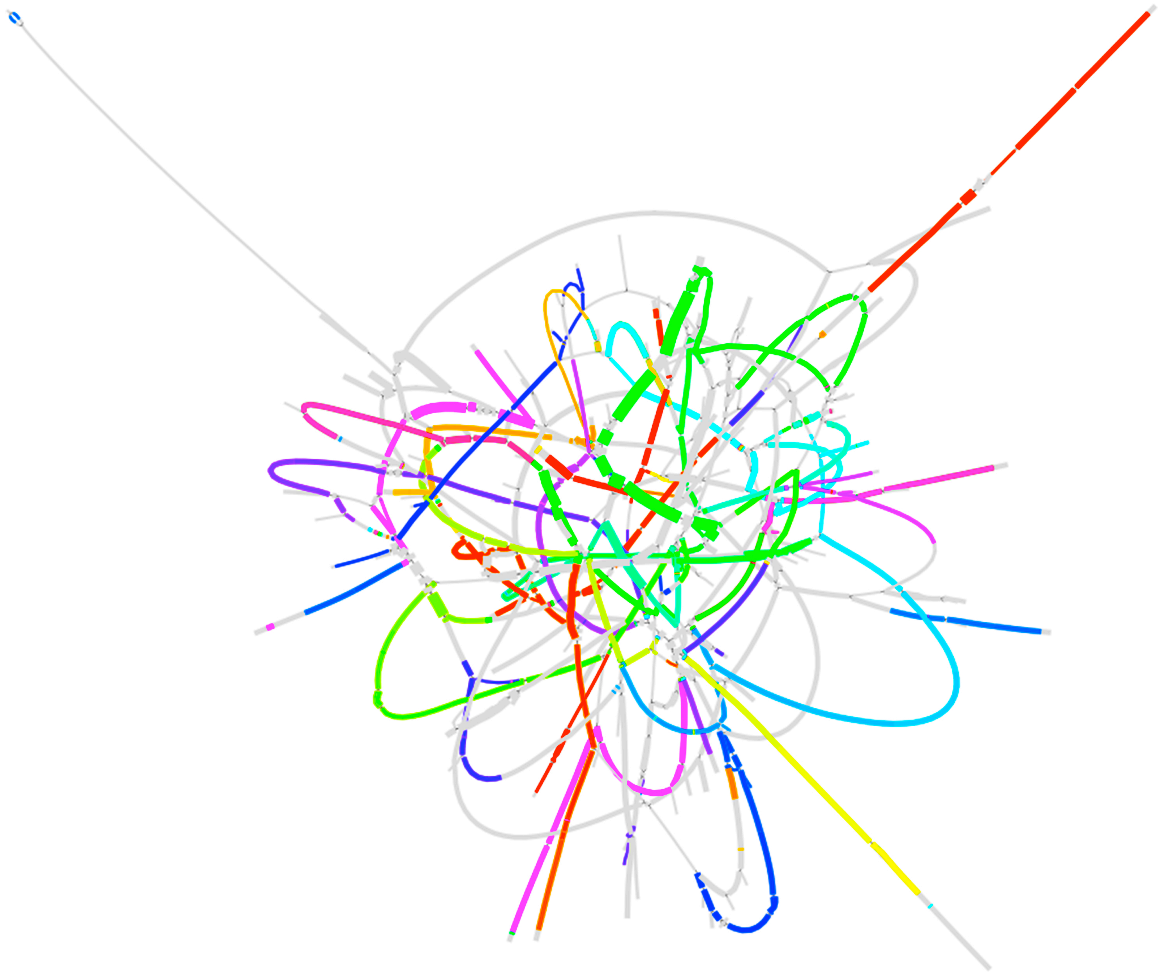

Assembly graph from SPAdes drawn with Bandage for the assembly of the *Miscanthus sinensis* cv Andante mitogenome. The *Saccharum* hybrid SP80-3280 mitochondrial chromosome 1 was mapped by BLAST and is shown in colour in the figure. Unmapped contigs are shown in grey. The sugarcane chromosome is both fragmented and has insertions as compared with the *M. sinensis* cv Andante assembly.

Figure 4. Assembly graph for the *Miscanthus sinensis* cv Andante mitogenome mapped by blast to the *Saccharum* hybrid SP80-3280 mitogenome chromosome 2.

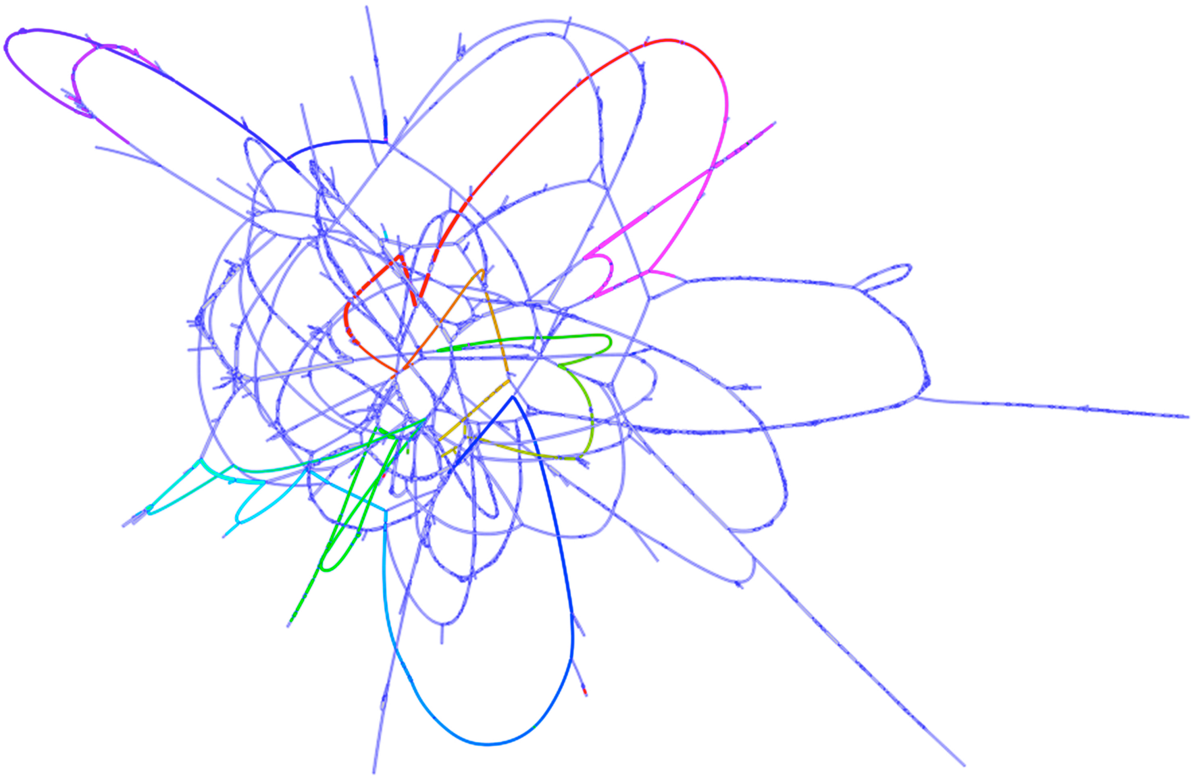

Assembly graph from SPAdes drawn with Bandage for the assembly of the *Miscanthus sinensis* cv Andante mitogenome. The *Saccharum* hybrid SP80-3280 mitochondrial chromosome 2 was mapped by BLAST and is shown in colour in the figure. Unmapped contigs are shown in grey. The sugarcane chromosome is both fragmented and has insertions as compared with the *M. sinensis* cv Andante assembly.

Comparisons of the two graphs above (Figure 3 and Figure 4) shows that there are separate contigs corresponding to the sugarcane mitochondrial chromosomes 1 and 2 in the *Miscanthus sinensis* cv Andante assembly graphs. However, the chromosomes as mapped are not contiguous and there are contigs joining them. Thus the structure of the *M. sinensis* mitogenome is different from that of the sugarcane mitogenome.

Comparing Figures 3 and 4 to Figures 1 and 2 reveals that the graphs for *S. spontaneum* and *M. sinensis* are quite similar, indicating that they share similar re-arrangements as compared with sugarcane.
