# Supplementary material for: The sugarcane mitochondrial genome: assembly, phylogenetics and transcriptomics
Source: PeerJ. 2019 Sep 24;7:e7558. doi: 10.7717/peerj.7558 (PMC6764373; doi:10.7717/peerj.7558)
Supplement: Supplemental Information 2 — Assembly graphs for the partial mitochondrial genome assemblies from Saccharum spontaneum SES234B and Miscanthus sinensis cv Andante showing overlapping BLAST mappings to the SP80-3280 mitochondrial chromosomes. [file peerj-07-7558-s002.pdf]

Origins and Analysis of Sugarcane CMS2 Transcript and Protein

Phylogenetically, the novel potential CMS factor (CMS2) identified in sugarcane is present in both the mitochondrial and nuclear (chromosome 9) genome of *Sorghum*. The two copies are divergent, thus the origin of the CMS remains uncertain. Most CMS factors are fusion proteins, typically emerging as combinations of existing mitochondrial genes/regions. Part of the sequence for this putative CMS was identified in the mitochondrial genome of *Tripsacum dactyloides*.

Using the sequences derived from *Saccharum* hybrid SP80-3280, *Sorghum bicolor* BTx623 and *Tripsacum dactyloides* the novel CMS factor was assembled for eight species (see below), To confirm the veracity of these assemblies primers were designed as it Table 1 and CMS regions were amplified and sequenced using ONT MinION (see Materials and Methods in the main manuscript for details).

Table 1

Species	Forward Primer	Reverse Primer	Tm (°C)	Amplicon (bp)
Miscanthus sinensis cv Andante	TCTTGGCAGCTCAAGACCTTTC	AGATCTCGGAAGAGCCGATGT	62.84	2877
Saccharum spontaneum SES234B	CAACTTTGAGTCGTATCCGTAAGAA	CCAACCGACGGGAGTTGTTAC	61	2812
Coix lacryma-jobi	CATCGGACTGAAAAGGAGGTC	CCAACCGACGGGAGTTGTTA	62.5	1954
Andropogon virginicus MT	ACATATAGAGGTCCTGCTTCTCC	CGAGTCAATCCTAGTAGAGAGAGG	57.95	2123
Andropogon virginicus G	TTTTATCTTTCATCAATCCCAAT	GCCCTCCTGTACAACCCAC	58	1266

Table 1 list of species, amplification primers, melting temperatures and amplicon lengths for the sequencing of CMS2 genes in four species.

Protein alignment analyses with protein domain overlay (Figure 1) demonstrates that the novel CMS factor s a combination of a non-cytoplasmc domain, a transmembrane domain corresponding to the N-terminus of mature nad6 and a cytoplasmic C-terminal domain.

Figure 1

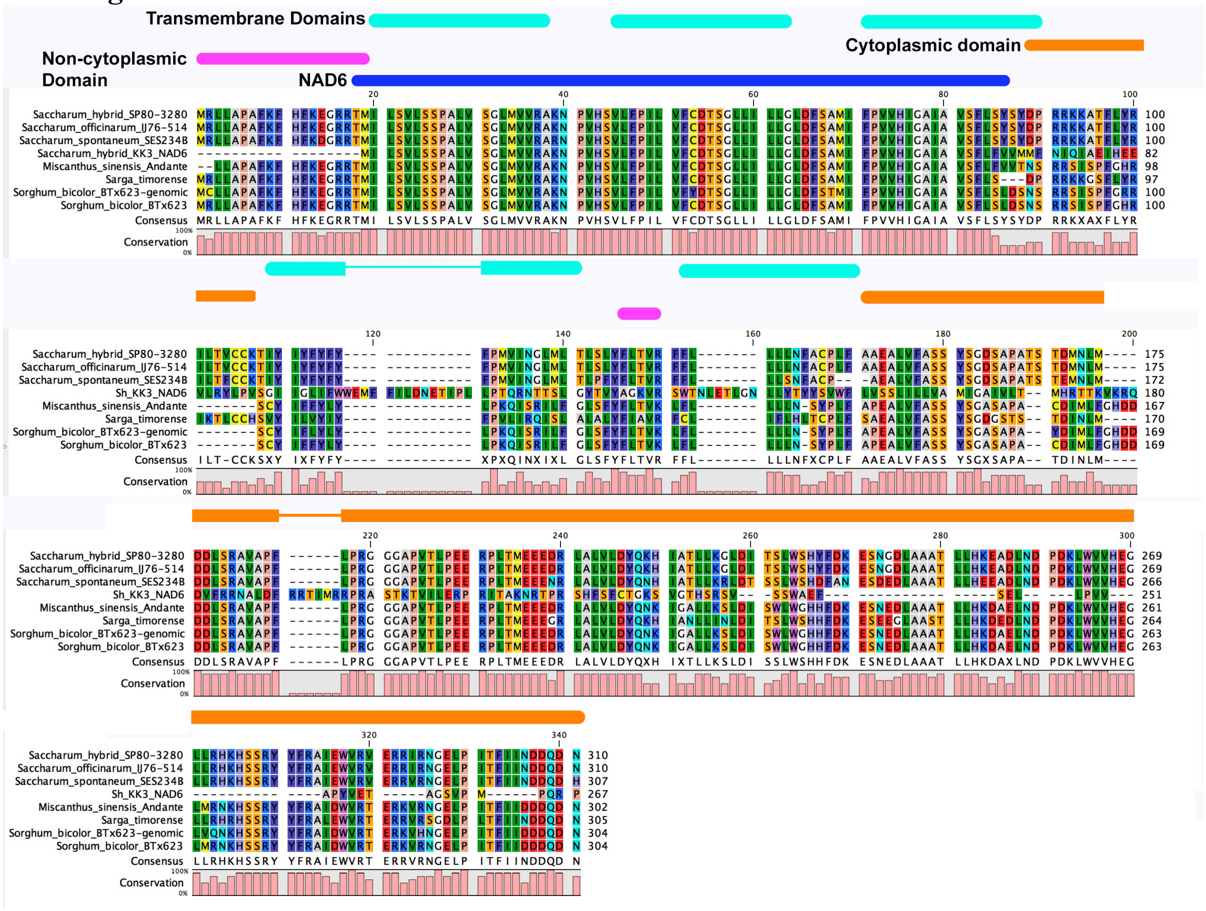

Multi-sequence alignment of the CMS2 gene in *Saccharum* hybrid, *Saccharum officinarum*, *Saccharum spontaneum*, *Miscanthus sinensis*, *Sarga timorense* and *Sorghum bicolor* mitochondrial genomes in comparison with the *Sorghum bicolor* genomic copy and the *Saccharum hybrid* mitochondrial copy of nad6. Overlaid on top is the InterProScan analysis of the domains present in the Sugarcane versions of CMS2.

Blast analyses of individual domains (N-terminal non-cytoplasmic, central nad6 and C-terminal cytoplasmic) revealed that the nad6 domain is present in mitochondrial nad6. The N-terminal domain is also present in nad6, but not normally transcribed and a sequence corresponding to part of the cytoplasmic domain was present in the mitochondrion of *Tripsacum dactyloides* but not the mitochondrion of *Zea mays*.

Chrysopogon zizanoides, in phylogenetic terms is the closest species (with sequence data available) to both *Tripsacum dactyloides* (where a partial C-terminus of CMS2 is present) and *Coix lacryma-jobi* (where a complete CMS2 is present). Only transcriptomic data was available, but assembly of the CMS2 C-terminal domain was possible (Figure 2).

Figure 2

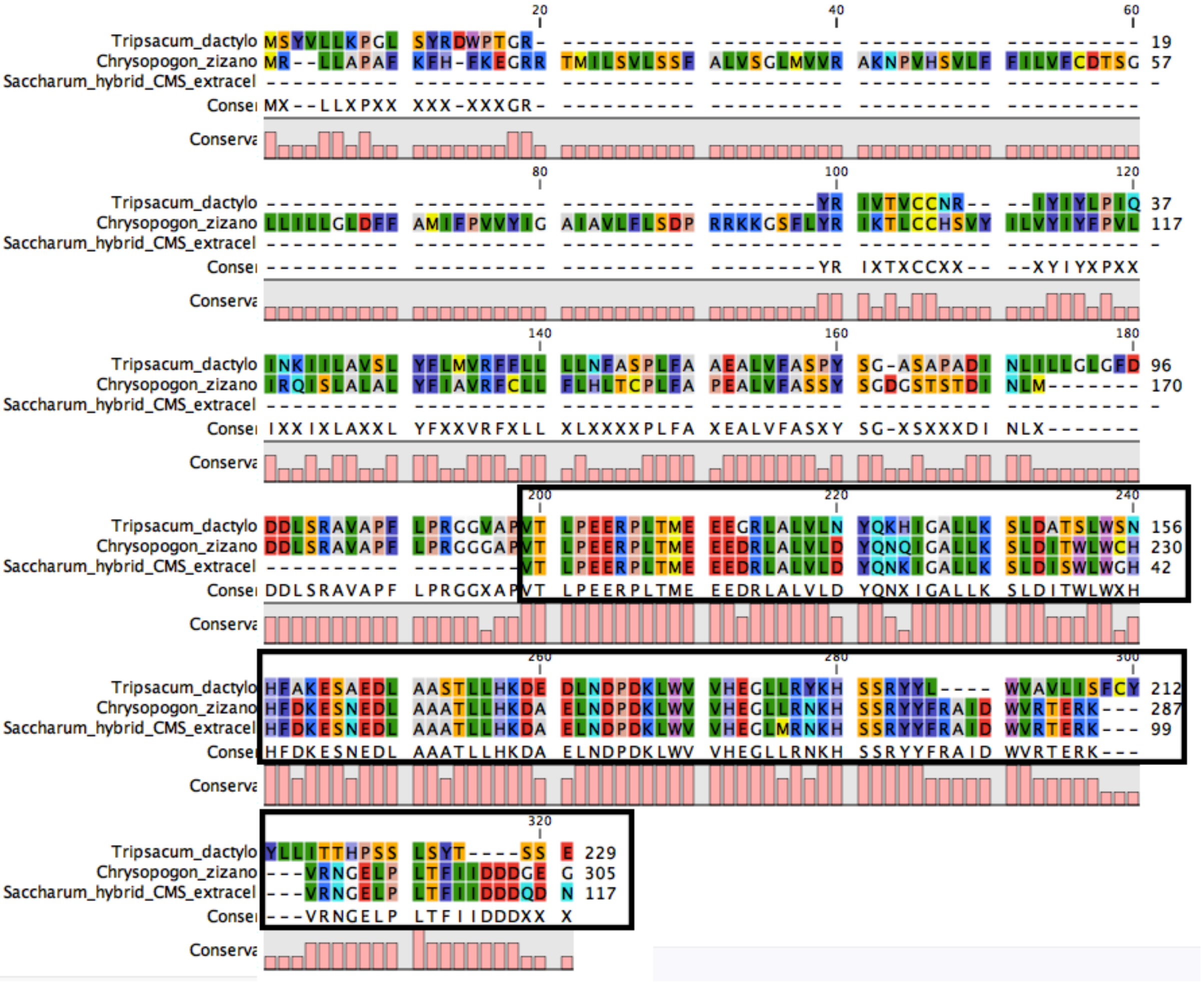

Alignmnt of the potential novel CMS genes from the mitochondria of *Chrysopogon zizanoides* and *Tripsacum dactyloides* with the cytosolic C-terminal domain of sugarcane CMS2. The boxed region represents the sequence corresponding to the C-terminus of the sugarcane protein.

In Figure 2, it can be shown that the protein in T. dactyloides does not contain a complete C-terminal domain as compared with sugarcane. However, C. zizanoides contains the complete C-terminal domain in conjunction with a partial nad6 N-terminal domain. Though this nad6 region is not the same as that in sugarcane.

The most parsimonious scenario is that the common ancestor of *C. zizanoides*, *T. dactyloides* and *Coix lacryma-jobi* had a nad6 – C-terminal cytoplasmic domain fusion. This made the 5' end of nad6 translateable. In *C. zizanoides* there were alterations to the nad6 and C-terminal domains. In *T. dactyloides* there were major alterations to the nad6 and C-terminal domains. In *Zea mays* the sequence was entirely lost.

This is shown schematically in Figure 3.

Figure 3

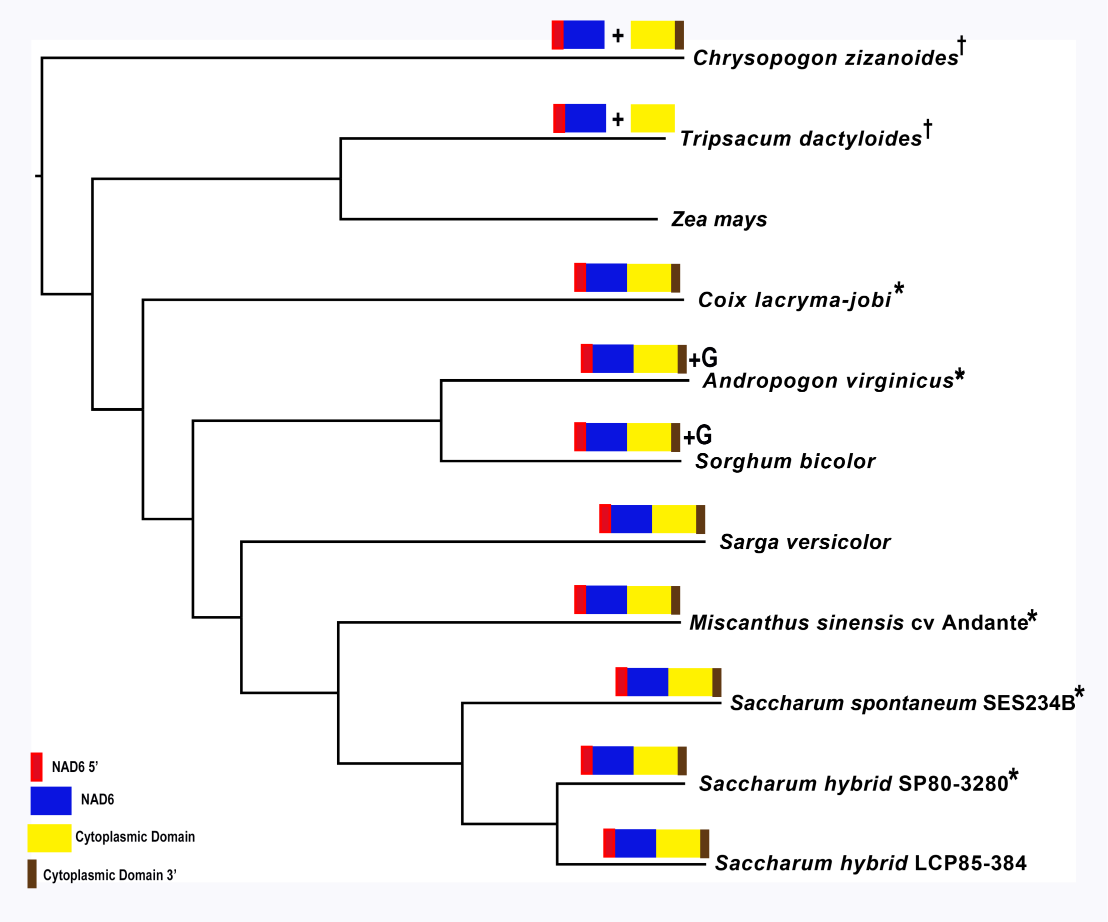

Phylogram based on Lloyd Evans et al. 2019, demonstrating the relationships between the genera *Saccharum*, *Miscanthus*, *Sarga*, *Sorghum*, *Andropogon*, *Zea*, *Tripsacum* and *Chrysopogon*. Components making up the new CMS2 in sugarcane are marked along with the evolutionary paths leading to them. G means a genomic as well as mitochondrial copy and '+' means that the components are part of multiple genes in that species.

Thus the origins of the components forming CMS2 in sugarcane are clear, as is the path to the loss of these components in Zea mays. Genomic and mitochondrial copies were found in both Sorghum bicolor and Andropogon virginicus, indicating the close relationship of the Sorghinae and the Core Andropogoneae.

Select species were sequenced to confirm the presence of the novel potential CMS in their mitogenomes. These are indicated with a * for genome data and † for transcriptomic data. Assembled mitochondrial regions are available from the Dryad digital repository (doi: https://doi.org/10.5061/dryad.634d24h) for assemblies from third party data and from ENA project (PRJEB32995) for directly sequenced data.

Thus the presence and evolutionary origins of CMS2 in sugarcane has been elucidated. However, this does not prove that the potential gene is functional and expressed. For this, transcriptomic analyses were performed comparing potentially novel genes against nad6 as a reference gene, which is known to be expressed, as well as a 1000bp non-coding region from the mitochondrial genome.

Table 2.

Spcies	Gene Name	Gene Length	Num Reads	Normalized Read Count
Chrysopogon zizanoides				
	nad6	1041	64	0.0615
	CMS2	918	1196	1.3028
	non-coding region	1000	22	0.022
	Fold Difference nad6	2.794		
	Fold Difference CMS2	59.22		
Tripsacum dactyloides				
	nad6	1016	3649	3.5915
	CMS21	639	63	0.0986
	non-coding region	1000	41	0.041
	Fold Difference nad6	87.59		
	Fold Difference CMS21	2.4		
Saccharum hybrid SP80-3280	nad6	804	23970	29.813
	CMS	345	1197	6.939
	CMS2	1175	15144	12.89
	non-coding region	1000	164	0.164
	Fold Difference nad6	181.79		
	Fold Difference CMS	42.312		
	Fold Dfference CMS2	78.59		
Sorghum bicolor BTx623				
	nad6	827	66	0.0798
	CMS2	940	73	0.0777
	non-coding region	1000	27	0.027
	Fold Difference nad6	29.55		
	Fold Dfference CMS2	28.777		

Determination of the expression potential of putative CMS genes. Raw reads from transcriptomic datasets for *Chrysopogon zizanoides*, *Tripsacum dactyloides*, *Saccharum* hybrid SP80-3280 and *Sorghum bicolor* were mapped with BWA against the putative CMS gene, nad6 gene and a non-coding mitochondrial region from each species. Total mapped reads (after duplicate removal) were normalized against the length of the CDS in bases and were expressed as fold change against the non-coding mitochondrial region.

Based on the results from Table 2, in *Chrysopogon zizanoides* there are significantly more transcripts for the CMS-like factor as compared with nad6 (over 21 fold more), a good indication that the CMS-like gene is transcribed. In contrast, the CMS-like gene in *Tripsacum dactyloides* is significantly truncated and is hardly expressed more than background (and significantly less than nad6), indicating that it may not be transcribed at all.

In *Sorghum bicolor*, the novel CMS2 gene is transcribed at almost the same level as nad6, indicating that it is a functional gene with real supporting transcripts.

In sugarcane, the first CMS factor, CMS is transcribed significantly above background (78-fold more) but only at 23% the level of nad6, whilst the second CMS, CMS2 is expressed at 40% of nad6. This is good support for both CMS factors being expressed as functional genes.
