# Supplementary material for: The sugarcane mitochondrial genome: assembly, phylogenetics and transcriptomics
Source: PeerJ. 2019 Sep 24;7:e7558. doi: 10.7717/peerj.7558 (PMC6764373; doi:10.7717/peerj.7558)
Supplement: Supplemental Information 3 — Mapping of RNA-seq reads to the multi-chromosomal mitogenomes of three species. Document gives column graphs of site by site mappings of RNA-seq reads to each chromosome in the multi-chromosome mitogenomes of Silene vulgaris, Cucumis sativus and Allium cepa showing almost complete coverage of each chromosome. The document lists all the SRA datasets employed in each of the mappings. [file peerj-07-7558-s003.pdf]

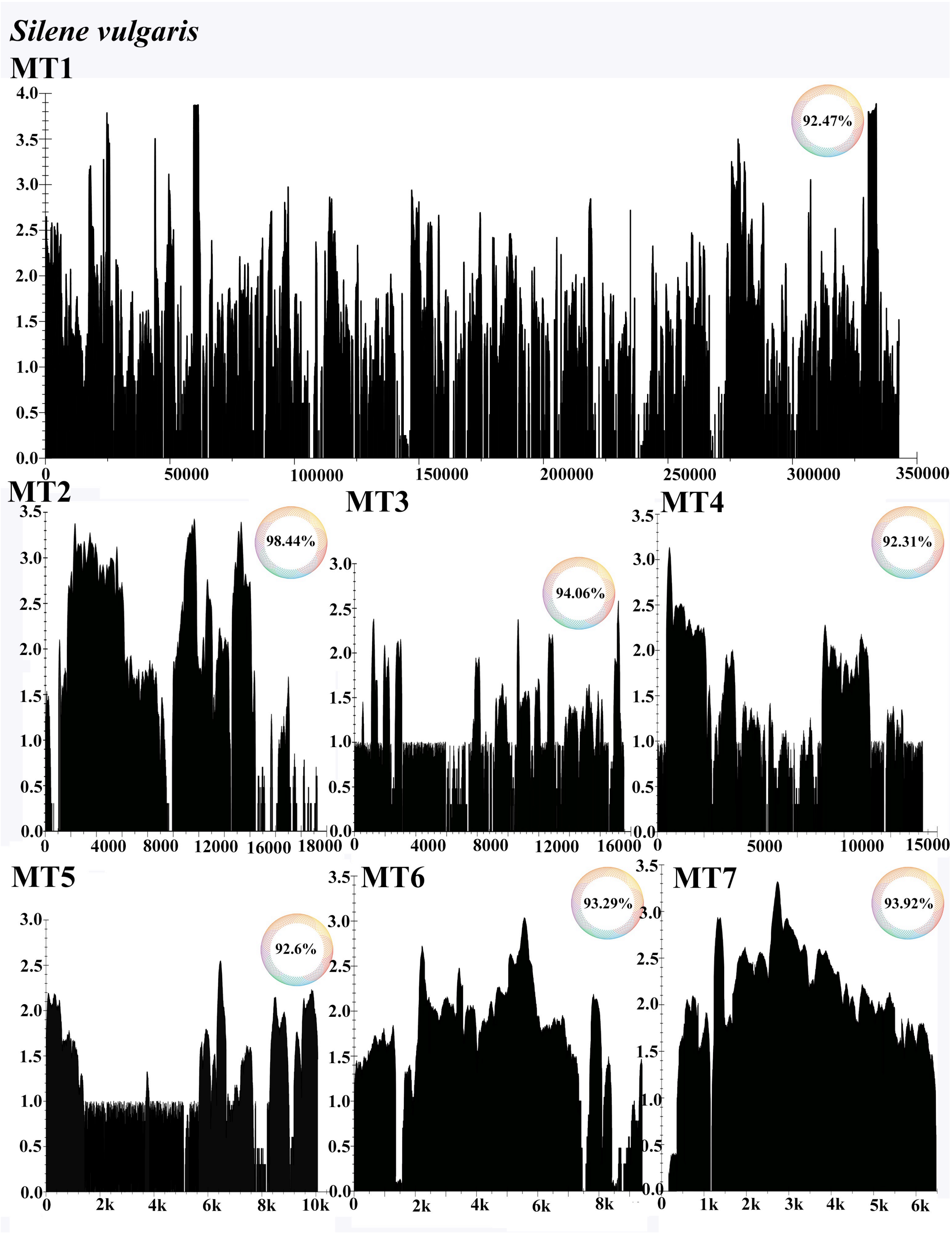

Mapping of SRA transcriptomic reads from *Silene vulgaris* to the seven mitochondrial chromosomes of the plant. The SRA datasets mapped were: SRR2106277; SRR2106278; SRR2106279; SRR342041; ERR142350; ERR1425350; ERR2356579; SRR1536113; SRR1536178; SRR245489 and SRR096785. In all cases read mapping to the individual chromosomes provided > 92% coverage, indicating that all the chromosomes and transcribed in their entirety.

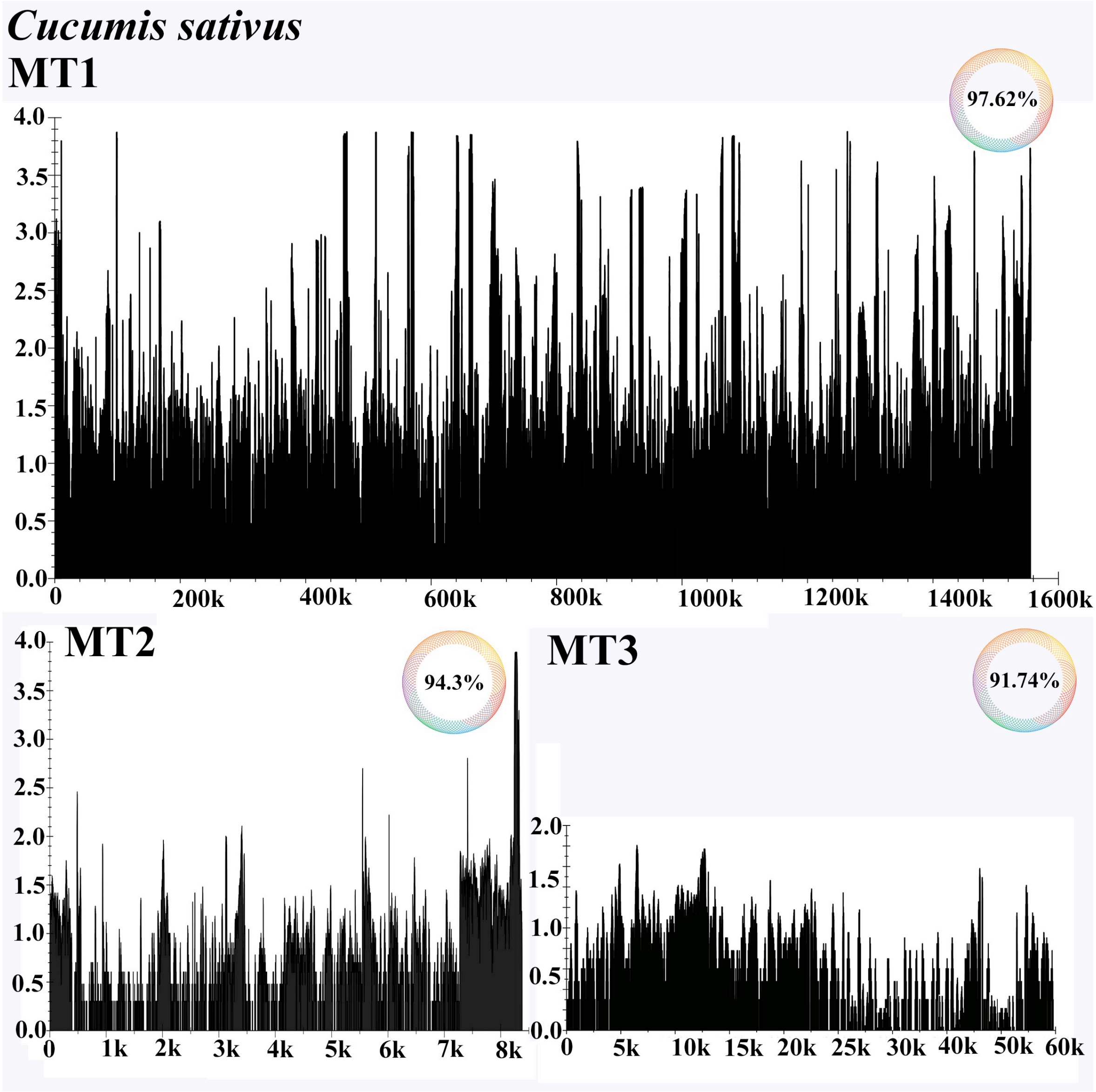

Mapping of SRA transcriptomic reads from *Cucumis sativus* to the three mitochondrial chromosomes of the plant. The SRA datasets mapped were: SRR6418022; SRR6650087; SRR6837824; SRR6957092; SRR7609886; SRR7609893; ERR662024; ERR662030; SRR2157748; SRR351499; SRR494480; SRR518995; SRR518990; SRR1548540, SRR1104924, SRR1104921, SRR1104922 and SRR1104901. In this instance there was >91.74% coverage of each mitochondrial chromosome from RNA-seq data, again indicating that each chromosome is transcribed in its entirety.

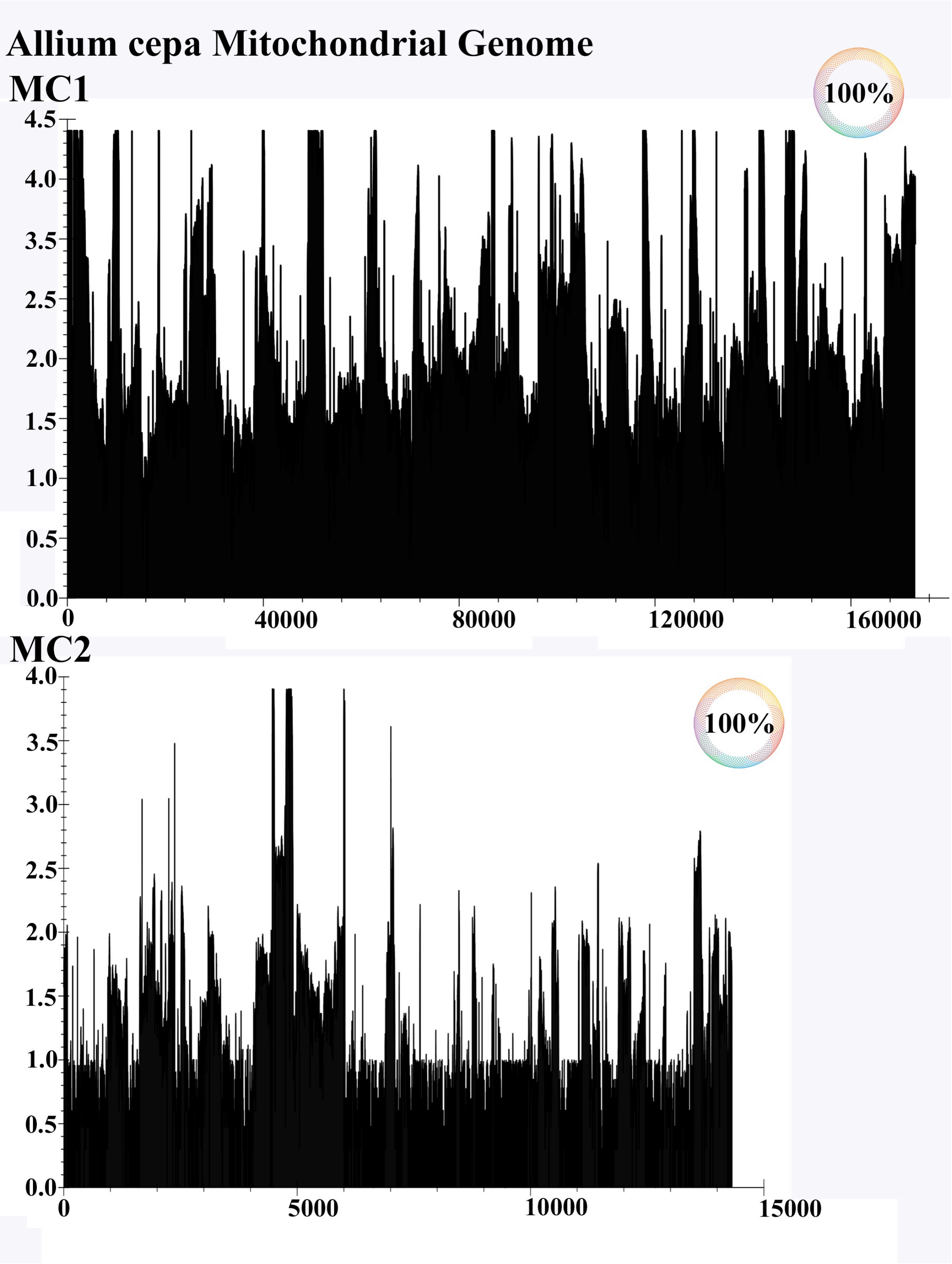

Mapping of SRA transcriptomic reads from *Allium cepa* to the two mitochondrial chromosomes of the male sterile plant. The SRA datasets mapped were: SRR1056445; SRR1958737; SRR2814816; SRR4418767; SRR073446; SRR4418768; SRR4418769; DRR068967; DRR068968; DRR068969; DRR068994; DRR068995 and DRR068996. In this case the two maxicircles of the mitogenome had 100% transcript coverage, demonstrating that they are both transcribed in their entirety.
