# Supplementary material for: The sugarcane mitochondrial genome: assembly, phylogenetics and transcriptomics
Source: PeerJ. 2019 Sep 24;7:e7558. doi: 10.7717/peerj.7558 (PMC6764373; doi:10.7717/peerj.7558)
Supplement: Supplemental Information 4 — Alignment of the rbcL protein from four sugarcane cultivars in comparison with the equivalent protein from S. spontaneum, S. officinarum and the sugarcane cultivar SP80-3280 chloroplast genome. [file peerj-07-7558-s004.pdf]

Supplemenal Document S4 — Protein sequence comparisons of rbcL from sugarcane chloroplas and mitochondrial assemblies.

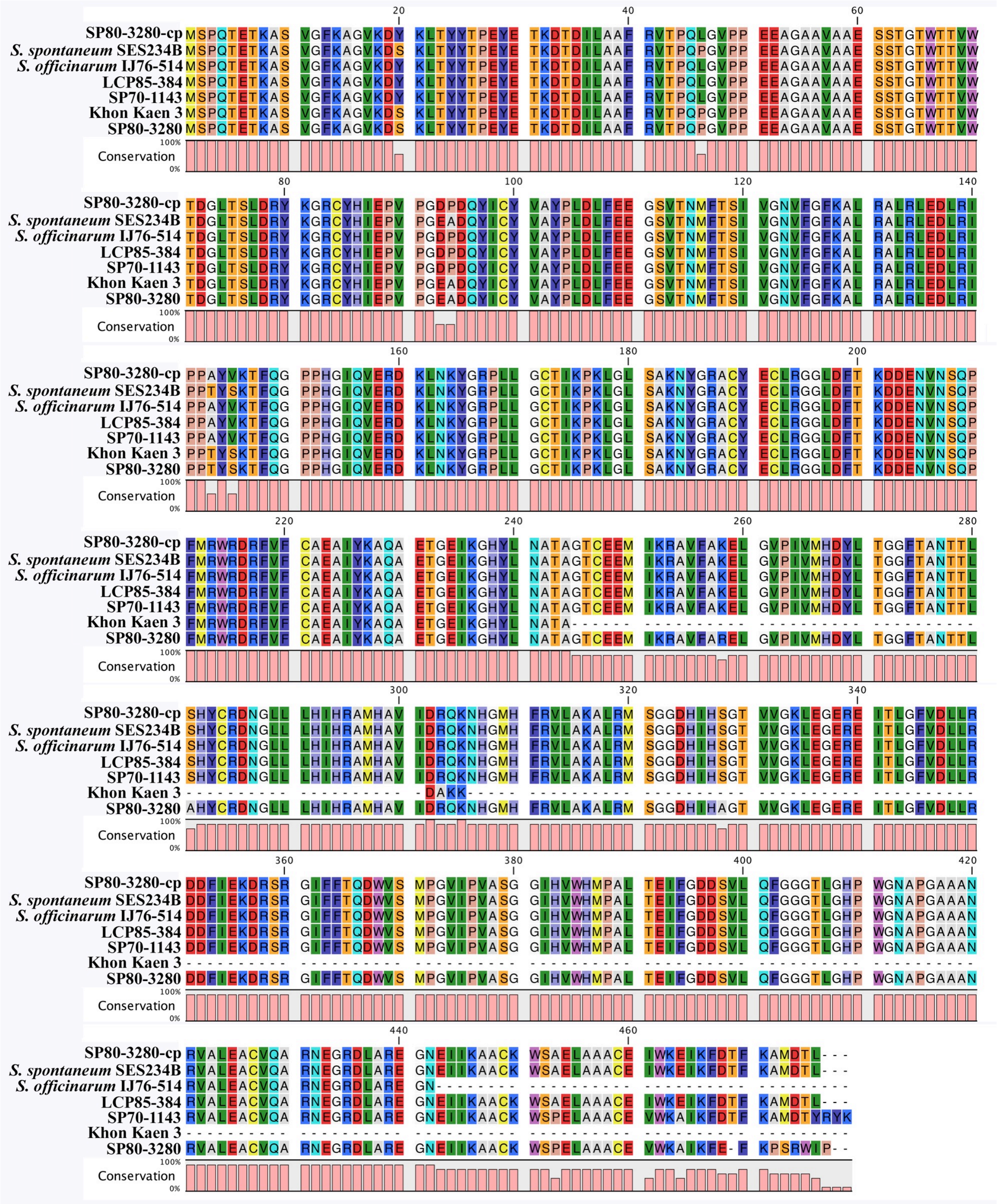

Alignment of the rbcL (Ribulose bisphosphate carboxylase large chain precursor) protein sequences from mitochondrial assemblies of the sugarcane cultivars LCP85-383, SP70-1143, Khon Kaen 3 and SP80-3280 along with the mitochondrial assemblies of *Saccharum spontaneum* SS234B and *S. officinarum* IJ76-514. All assemblies are aligned to the

chloroplast sequence of rbcL from SP80-3280 (labelled SP80-3280-cp). Alignments were performed with Saté and exported from CLC Sequence Viewer v. 7.
