# Supplementary material for: The sugarcane mitochondrial genome: assembly, phylogenetics and transcriptomics
Source: PeerJ. 2019 Sep 24;7:e7558. doi: 10.7717/peerj.7558 (PMC6764373; doi:10.7717/peerj.7558)
Supplement: Supplemental Information 5 — A complete listing of splice sites in the sugarcane mitochondrial genome. A listing of all splice sites with occurrence >10 in the sugarcane mitochondrial genome. The table lists the splice sites positions, upstream and downstream genes and the notes give any coding sequences that the splice sites lie within. [file peerj-07-7558-s005.docx]

Supplemental Table S1

| **Species** | **Voucher or Accession** | **GenBank**  **Accession** | | **Reference** | | **GC Content (%)** | |
| --- | --- | --- | --- | --- | --- | --- | --- |
| **Chloroplast** |  |  |  | |  | |  |
| *Sorghum bicolor* | BTx623 | EF115542.1 | Saski et al. 2007 | | 38.5 | |  |
| *Sarga timorense* |  | KF998272.1 | Kepers *et al*. (submitter) | | 38.5 | |  |
| *Miscanthidium junceum* |  | LN869216 | Lloyd Evans, D. (submitter) | | 38.4 | |  |
| *Miscanthidium capense* | SASRI:Joshi1 | PRJEB17861 | Lloyd Evans, D. (submitter) | | 38.4 | |  |
| *Miscanthus floridulus* | PI295762 | LN869215.1 | Lloyd Evans, D. (submitter) | | 38.4 | |  |
| *Miscanthus sinensis* | Andante | LM735682 | Lloyd Evans, D. (submitter) | | 38.4 | |  |
| *Saccharum* hybrid | SP80-3280 | AE009947.2 | Calsa Jr *et al*. 2004 | | 38.4 | |  |
| *Saccharum* hybrid | SP80-3280 (transcriptomic) | Ibid |  | | 38.4 | |  |
| *Saccharum* hybrid | Q165 | LN896359.1 | Lloyd Evans and Joshi 2016 | | 38.4 | |  |
| *Saccharum* hybrid | SP70-1143 | Ibid |  | | 38.4 | |  |
| *Saccharum officinarum* | IJ76-514 | LN849913 | Lloyd Evans and Joshi 2016 | | 38.4 | |  |
| *Saccharum spontaneum* | SES196 | PRJEB20532 | Lloyd Evans et al. 2019 | | 38.5 | |  |
| *Saccharum spontaneum* | SES234B | LN849912.1 | Lloyd Evans, D. (submitter) | | 38.4 | |  |
| *Zea mays* | B73 | AY928077.1 | Schnable et al. 2009 | | 38.5 | |  |
| *Miscanthus sacchariflorus* | Hercules | LN869218.1 | Lloyd Evans, D. (submitter) | | 38.4 | |  |
|  |  |  |  | |  | |  |
| **Mitochondria** |  |  |  | |  | |  |
| *Saccharum* hybrid | Khon Kaen | LC107874.1 LC107875.1 | Shearman *et al*. 2016 | | 43.82 | |  |
| *Saccharum* hybrid | LCP85-384 | ibid |  | | 43.82 | |  |
| *Saccharum* hybrid | RB72454 | Ibid |  | | 43.82 | |  |
| *Saccharum* hybrid | SP70-1143 | Ibid |  | | 43.83 | |  |
| *Saccharum* hybrid | R570 | Ibid |  | | 43.83 | |  |
| *Saccharum officinarum* | IJ76-513 | Ibid |  | | 43.07 | |  |
| *Sorghum bicolor* | BTx623 | DQ984518.1 | Allen et al. 2007 | | 43.73 | |  |
| *Tripsacum dactyloides* | Pete | NC_008362.1 | Allen et al. 2007 | | 43.93 | |  |
| *Zea mays* | mays | NC_007982.1 | Clifton et al. 2004 | | 43.93 | |  |
| *Zea perennis* |  | DQ645538.1 | Allen et al. 2007 | | 43.88 | |  |
|  |  |  |  | |  | |  |
| **Whole Genome** |  |  |  | |  | |  |
| *Sorghum bicolor* | BTx623 |  | Paterson et al. 2008 | | 41.4 | |  |
| *Zea mays* | B73 |  | Schnable et al. 2009 | | 47.2 | |  |
| *Saccharum* hybrid | SP80-3280 | SRA:SRR1763296 |  | | 42.7 | |  |

Species name, voucher accession, GenBank accession and reference for plastomes, mitochondria and whole genomes analysed for GC content in this study. The final column gives the percentage GC content for the respective genome. The SRA accession is for the *Saccharum* hybrid SP80-3280 illumina virtual long read dataset.

References:

Allen, J.O., Fauron, C.M., Minx, P., Roark, L., Oddiraju, S., Lin, G.N., Meyer, L., Sun, H., Kim, K., Wang, C. and Du, F., 2007. Comparisons among two fertile and three male-sterile mitochondrial genomes of maize. Genetics, 177:1173–1192.

Calsa Jr T, Carraro DM, Benatti MR, Barbosa AC, Kitajima JP, Carrer H. 2004. Structural features and transcript-editing analysis of sugarcane (Saccharum officinarum L.) chloroplast genome. Current Genetics 46:366–373.

Clifton, S.W., Minx, P., Fauron, C.M.R., Gibson, M., Allen, J.O., Sun, H., Thompson, M., Barbazuk, W.B., Kanuganti, S., Tayloe, C. and Meyer, L., 2004. Sequence and comparative analysis of the maize NB mitochondrial genome. Plant Physiology, 136:3486–3503.

Lloyd Evans D, Joshi SV. 2016. Complete chloroplast genomes of Saccharum spontaneum, Saccharum officinarum and Miscanthus floridulus (Panicoideae: Andropogoneae) reveal the plastid view on sugarcane origins. Systematics and Biodiversity, 14:548–571.

Lloyd Evans D, Joshi SV, Wang J. 2019. Whole chloroplast genome and gene locus phylogenies reveal the taxonomic placement and relationship of *Tripidium* (Panicoideae: Andropogoneae) to sugarcane. *BMC Evolutionary Biology*19:33.

Paterson, A.H., Bowers, J.E., Bruggmann, R., Grimwood, J., Gundlach, H., Haberer, G., Hellsten, U., Mitros, T., Poliakov, A., Schmutz, J. and Spannagl, M., 2008. The Sorghum bicolor genome and the diversification of grasses. Nature, 457(LBNL-6812E).

Saski C, Lee SB, Fjellheim S, Guda C, Jansen RK, Luo H, Tomkins J, Rognli OA, Daniell H, Clarke JL. 2007. Complete chloroplast genome sequences of Hordeum vulgare, Sorghum bicolor and Agrostis stolonifera, and comparative analyses with other grass genomes. Theoretical and Applied Genetics 115:571–590.

Schnable PS, Ware D, Fulton RS, Stein JC, Wei F, Pasternak S, Liang C, Zhang J, Fulton L, Graves TA, Minx P. 2009. The B73 maize genome: complexity, diversity, and dynamics. Science 326:1112–1115.

Shearman, J.R., Sonthirod, C., Naktang, C., Pootakham, W., Yoocha, T., Sangsrakru, D., Jomchai, N., Tragoonrung, S. and Tangphatsornruang, S., 2016. The two chromosomes of the mitochondrial genome of a sugarcane cultivar: assembly and recombination analysis using long PacBio reads. Scientific Reports, 6:31533.
