# Supplementary material for: The sugarcane mitochondrial genome: assembly, phylogenetics and transcriptomics
Source: PeerJ. 2019 Sep 24;7:e7558. doi: 10.7717/peerj.7558 (PMC6764373; doi:10.7717/peerj.7558)
Supplement: Supplemental Information 6 — Summary of GC content in chloroplast, mitochondrial and nuclear genomes of a representative sample of Sacchainae. Table headers represent: species, species name; voucher or accession, species voucher, cultivar name or accession; GenBank accession, GenBank identifier of sequence (if available); reference, published reference for the sequence and GC Content (%), percentage GC content in the genome. [file peerj-07-7558-s006.docx]

Supplemental Table S2 — Summary of GC content in chloroplast, mitochondrial and nuclear genomes of a representative sample of Sacchainae

| **Splice Sites** | |  |  |  | | | |  |
| --- | --- | --- | --- | --- | --- | --- | --- | --- |
|  |  |  |  |  | | | |  |
| **Start** | **End** | **Strand** | **Gene 1** | | | **Gene2** | | **Notes** |
| **MT1** |  |  |  | | |  | |  |
| 1104 | 98718 | plus | mttB | | | atp8 | | Second splice junction is in the middle of ccmC |
| 7046 | MT2:8786 | plus | mttB | | | ccmFc | | first splice lies in atp1; Second splice junction lies within rrn26 |
| 8267 | 80310 | minus | mttB | | | trmL | | Second splice junction lies in a nad7 exon may splice with putative protein OQU77742 |
| 8600 | 40833 | minus | mttB | | | nad7 | | First splice site in atp1, second splice iste in rps2 (end) |
| 8600 | 99084 | minus | mttB | | | atp8 | | Second splice site in rrn26 |
| 33911 | 60869 | plus | trnY | | | nad7 ex2/trnS | | |
| 36771 | 61377 | minus | trnY | | | nad7 ex2/trnS | | second splice junction in middle or rrn18 |
| 37053 | 101043 | minus | trnY | | | atp8 | | second splice juncion lies within rrn26 |
| 38863 | 109527 | plus | trnY | | | rrn5/cox1 | | Second splice junction lies within rrn26 |
| 45823 | 63316 | minus | rps2 | | | trnS | |  |
| 45823 | 98028 | minus | rps2 | | | atp8 | | Second splice junction lies within rrn26 |
| 54613 | 110493 | minus | rps2 | | | rrn5/cox1 | | Second splice junction lies within rrn26 |
| 54613 | 60420 | plus | rps2 | | | nad7 exons 1 and 2/ trnS | | Second splice site lies within nad7 exon3 |
| 57286 | 59090 | plus | rps2 | | | nad 7 exons 1,2,3 | | First splice site lies within nad7 exon5, second splice lies in nad7 exon 4 |
| 57216 | 164873 | minus | rps2 | | | trnP | | First splice site lies within nad7 exon5, second splice lies in nad1 exon |
| 57348 | 59159 | plus | rps2 | | | nad7 exons 1,2,3 | | First splice site lies within nad7 exon5, second splice lies in nad7 exon 4 |
| 59231 | 60394 | plus | nad7 exon 5,rps2 | | | nad7-exon3 | | First splice site lies within nad7-exon4, second splice site lies within nad7 exon3 |
| 60629 | 62177 | minus | nad7 exon 4,5 | | | nad7-exon1 | | First splice site lies within nad7-exon 3 |
| 61355 | 98434 | plus | nad7 exons 3,4,5 | | | atp8 | | Second splice site in rrn18 |
| 62030 | 109496 | plus | nad7 exons 3,4,5 | | | rrn5/cox1 | | Second splice junction lies within rrn26 |
| 73158 | 82585 | minus | rps7 | | | rrn18 | |  |
| 73158 | 120822 | minus | rps7 | | | cox2 | |  |
| 73158 | 123280 | minus | rps7 | | | trnI/transposon-derived Pol | | |
| 73158 | 157262 | minus | rps7 | | | nad6 |  | |
| 73158 | 163958 | minus | rps7 | | | nad1 internal/trnP | | |
| 81050 | 177766 | plus | ccmC | | | nad2 | |  |
| 92752 | 100956 | plus | ccmC | | | atp8 | | second splice in rrn18 |
| 97213 | 113571 | plus | ccmC | | | cox2 | |  |
| 97213 | 123287 | minus | ccmC | | | trnI | |  |
| 97213 | 131450 | minus | ccmC | | | trnH | |  |
| 97213 | 159437 | minus | ccmC | | | nad1 interna exon/trnP | | second splice in nad6 |
| 98250 | 285335 | plus | ccmC | | | rrn18/atp8 | | first splice in rrn18, second splice in rrn18 |
| 98676 | 286736 | plus | ccmC | | | rrn18/atp8 | | first splice in rrn18, second splice in rrn18 |
| 98776 | 286551 | minus | ccmC | | | rrn18/atp8 | | first splice in rrn18, second splice in rrn18 |
| 99212 | 286859 | plus | ccmC | | | rrn18/atp8 | | first splice in rrn18, second splice in rrn18 |
| 99310 | 261289 | plus | ccmC | | | rrn18/atp8 | | first splice in rrn18, second splice in rrn18 |
| 100380 | 288506 | minus | ccmC | | | rrn18/atp8 | | first splice in rrn18, second splice in rrn18 |
| 102125 | 112393 | plus | rrn18 | | | cox2 | | first splice in atp8, second splice in cox1 |
| 110406 | 220357 | minus | atp8 | | | trnK | | first splice in rrn28, |
| 110652 | 296822 | minus | atp8 | | | rrn26, rrn5 | | first splice in rrn28, second splice in rrn26 |
| 111144 | 298842 | minus | rrn26 | | | rrn5, cox1 pseudo | | first splice in rrn5, second splice in rrn5 |
| 113091 | 300696 | plus | cox1 | | | nad5 (if spliced to mt2) or end of cox1 pseudogene | | |
| 113455 | 190188 | plus | cox1 | | | nad2 | |  |
| 113462 | MT2:49855 | plus | cox1 | | | rps12 | |  |
| 121227 | 122595 | plus | cox2 exon1 | | | cox2 exon2 | | second splice within cox2 exon2 but product translates into a truncated cox II |
| 122828 | 286711 | minus | cox2 exon1 | | | atp8 | | first intron within cox 2 exon2, second exon within rrn18 |
| 132061 | 132849 | minus | trnI | | | trnH | |  |
| 133416 | MT2:50053 | plus | trnH | | | rps12 | |  |
| 137290 | 164839 | minus | ccmB | | | trnP | | second splice within exon of nad1 |
| 148224 | 149756 | minus | trnP | | | rps13 | | first splice within exon of nad1, second splice within exon of nad1 |
| 149730 | 164836 | plus | trnP | | | trnP, nad1 | | first splice within exon of nad1, second splice within exon of nad1 |
| 149764 | 164670 | plus | trnP | | | trnP, nad1 | | first splice within exon of nad1, second splice within exon of nad1 |
| 149765 | 168871 | plus | trnP | | | nad2 | | first splice within exon of nad1, |
| 152961 | 181473 | plus | rps13 | | | nad2 | |  |
| 156010 | 208774 | minus | rps13 | | | nad5 | | first splice within nad4 |
| 159327 | MT2:26275 | minus | atp4 | | | trnM | | first splice within nad6 |
| 190228 | 240235 | plus | trnP | | | matR | | second splice in last exon of nad1 |
| 190260 | 266329 | minus | trnP | | | trnS | |  |
| 190316 | MT2:107755 | plus | trnP | | | cob | |  |
| 190334 | MT2:107822 | minus | trnP | | | cob | |  |
| 190410 | 286763 | minus | trnP | | | atp8 | | second splice in rrn28 |
| 191530 | 192476 | minus | trnP |  | | | | first splice in nad2 exon1 |
| 192363 | MT2:49741 | plus | trnP, nad2 exon1 | | | | | first splice in nad2 exon2 |
| 208541 | 209534 | minus | nad2 exon1 and 2 | | trnK | | | first splice in nad5 exon1, second splice in nad5 exon1 |
| 213190 | 221562 | minus | nad5 exons 1 and 2 | | trnK | | | second splice site in nad2 exon2 |
| 227905 | MT2:27085 | minus | nad5 exons 1 and 2 | | trnM | | |  |
| 228357 | 287403 | minus | nad5 exons 1 and 2 | | atp8 | | | second splice in rrn28 |
| 228406 | 298127 | minus | nad5 exons 1 and 2 | | atp8 | | | second splice in rrn28 |
| 228406 | 246601 | minus | nad5 exons 1 and 2 | | ccmFn | | | second splice in rps1 |
| 228357 | 286902 | minus | nad5 exons 1 and 2 | | atp8 | | | second splice in rrn28 |
| 228357 | 299492 | minus | nad5 exons 1 and 2 | | nad5 (if spliced to  mt2) or end of cox1  pseudogene | | | |
| 229690 | 286916 | minus | nad5 exons 1 and 2 | | atp8 | | | second splice in rrn28 |
| 229690 | 298056 | plus | nad5 exons 1 and 2 | | rrn5 | | | second splice in rrn18 |
| 229690 | MT2:26347 | minus | nad5 exons 1 and 2 | | trnM | | |  |
| 260131 | MT2:10860 | minus | trnE | | ccmFc | | |  |
| 285490 | MT2:49914 | plus | trnN | | rps12 | | | first splice in rrn28 |
| 285490 | MT2:136773 | plus | trnN | | nad 4 exons 3 and 4 | | | first splice in rrn28 |
| 286104 | MT2:121378 | minus | trnN | | nad4 | | | first splice in rrn28, second splice cite in cob gene |
| 286891 | MT2:15424 | minus | trnN | | trnM | | | first splice in rrn28 |
| 287271 | 298036 | plus | trnN | | rrn5 | | | first splice in rrn28, second splice in rrn16 |
| 287410 | MT2:49757 | plus | trnN | | rps12 | | | first splice in rrn28, second splice in atp6 |
| 287410 | MT2:49747 | minus | trnN | | rps12 | | | first splice in rrn28, second splice in atp6 |
| 287809 | MT2:98682 | minus | trnN | | cob | | | first splice in rrn28 |
| 289893 | MT2:51 | minus | rrn26 | | nad5 | | | first splice in atp8 |
| 298262 | MT2:40031 | plus | atp8 | | atp6 | | | first splice in rrn16 |
| 299064 | MT2:49741 | plus | rrn5 | | rps12 | | | second splice site in atp6 |
| 299235 | MT2:49808 | minus | rrn5 | | rps12 | | | second splice site in atp6 |
| 299235 | MT2:49976 | minus | rrn5 | | rps12 | | |  |
| 300617 | MT2:126830 | plus | rrn5 | | nad4 | | |  |
|  |  |  |  | |  | | |  |
| **MT2** |  |  |  | |  | | |  |
| 9579 | 10497 |  | trnC-GCA | | rps11-cp | | | second splice site in petD-cp |
| 2821 | 55961 |  |  | | nad4-ex1 | | | first splice site in rps4 |
| 9616 | 10463 |  | trnC-GCA | | trnM-CAT | | |  |
| 10992 | 49222 |  | petD-cp | | cob | | |  |
| 12294 | 54643 |  | petD-cp | | nad4-ex1 | | | first splice site in rps11-cp, second splice site in cob |
| 19608 | 54949 |  | nad2-ex4 | | nad4-ex1 | | |  |
| 19833 | 21789 |  | nad2-ex3 | | trnM-CAT | | | first splice in nad2-ex4, second splice in nad2-ex3 |
| 21963 | 48957 |  | trnM-CAT | | cob | | |  |
| 49192 | 49744 |  | nad4L | | cob | | | second splice site in psbA-cp-pseudo |
| 49633 | 50089 |  | nad4L | | cob | | | first splice site in psbA-cp-pseudo, second splice site in psbA-cp-pseudo |
| 49653 | 49976 |  | nad4L | | cob | | | first splice site in psbA-cp-pseudo, second splice site in psbA-cp-pseudo |
| 49790 | 136771 |  | nad4L | |  | | | first splice site in psbA-cp-pseudo, second splice site in cox3 |
| 49997 | 97802 |  | nad4L | | trnM-CAT | | | first splice site in psbA-cp-pseudo |
| 53730 | 107969 |  | nad4L | | atp9 | | | first splice site in cob |
| 64524 | 88678 |  | cob | | ccmFc-ex2 | | | first splice site in nad4-ex1, seond splice site in ccmFc-ex1 |
| 66727 | 70305 |  | nad4-ex1 | | nad4-ex4 | | | first splice site in nad4-ex2, seond splice site in nad4-ex3 |
| 70574 | 73358 |  | nad4-ex2 | | orf49 | | | first splice site in nad4-ex3, second splice site in nad4-ex4 |
| 71136 | 77358 |  | nad4-ex3 | | nad5-ex4 | | |  |
| 82589 | 83642 |  | orf49-cp | | orf104 | | | first exon in nad5-ex4, second exon in nad5-ex5 |
| 107825 | 136241 |  | trnM-CAT | | cox3 | | |  |
| 114871 | 116891 |  | rpl16 | | rps12 | | | first exon in rps3-ex2 |
| 133997 | 138016 |  | trnS-GCT | |  | | | first exon in atpA-cp |

Summary of GC content in chloroplast, mitochondrial and nuclear genomes of a representative sample of Sacchainae. Table headers represent: species, species name; voucher or accession, species voucher, cultivar name or accession; GenBank accession, GenBank identifier of sequence (if available); reference, published reference for the sequence and GC Content (%), percentage GC content in the genome.
