# Supplementary material for: The sugarcane mitochondrial genome: assembly, phylogenetics and transcriptomics
Source: PeerJ. 2019 Sep 24;7:e7558. doi: 10.7717/peerj.7558 (PMC6764373; doi:10.7717/peerj.7558)
Supplement: Supplemental Information 7 — Table listing polyA baited read mapping to the Sorghum bicolor BTx623 mitochondrial genome. Table lists the start and end of all contiguous polyA read mapping sites with depth >500 reads along with any genes or notable featured within the mapping region. [file peerj-07-7558-s007.docx]

Supplemental Table S3 — PolyA baited read mapping to the Sorghum bicolor BTx623 mitochondrial genome

| **region number** | **start** | **end** | **annotation** | **region length** | | | |  |
| --- | --- | --- | --- | --- | --- | --- | --- | --- |
| 1 | 7 | 886 | mttb-1 | 880 |  | | | |
| 2 | 2219 | 3251 | atpB chloroplast | 1033 |  | | | |
| 3 | 6548 | 8141 | rpoc3 cp pseudo | 1594 |  | | | |
| 4 | 12015 | 15137 | atp1-1 | 3123 |  | | | |
| 5 | 21824 | 26274 | nad1 exons 2 &3 | 4451 |  | | | |
| 6 | 48078 | 51483 | trnY nad9 trnF | 3406 |  | | | |
| 7 | 66272 | 68425 | trnS, trnL-b,nad3, rps12 | 2154 |  | | | |
| 8 | 70094 | 71263 | vesicle associated protein 2 exon 1 (nuclear) | 1170 |  | | | |
| 9 | 74036 | 76534 | rps2B, nad1 | 2499 |  |  |  |  |
| 10 | 82310 | 84731 | mt intron | 2422 |  |  |  |  |
| 11 | 92057 | 94240 | rpoc2 cp fragment 2 pseudogene | 2184 |  | | | |
| 12 | 118722 | 121479 | cox3, nad1 (exon) | 2758 |  | | | |
| 13 | 125976 | 129040 | ATP synthase pseudogene | 3065 |  | | | |
| 14 | 138663 | 141806 | rps13 | 3144 |  | | | |
| 15 | 159759 | 162629 | rps1, ccmFN | 2871 |  | | | |
| 16 | 182051 | 184133 | nad7 ex 2 | 2083 |  | | | |
| 17 | 195082 | 197502 | cob-1 | 2421 |  | | | |
| 18 | 202452 | 203673 | part of 1.9kb repeat, copy 1 | 1222 |  | | | |
| 19 | 221007 | 226547 | rrn26, atp8 | 5541 |  | | | |
| 20 | 228874 | 229061 | conserved small repeat | 188 |  | | | |
| 21 | 232514 | 235697 | rrn18, rrn5 | 3184 |  | | | |
| 22 | 250130 | 254235 | rps8-cp, rpl4-cp, rpl16-cp pseudogene | 4106 |  | | | |
| 23 | 290341 | 294508 | nad5 ex 2, 3 | 4168 |  | | | |
| 24 | 297583 | 298204 | rps16-cp intron | 622 |  | | | |
| 25 | 306860 | 307579 | nad1 intron | 720 |  | | | |
| 26 | 312617 | 318910 | trnP-b-cp, trnW-cp, ccmB | 6294 |  | | | |
| 27 | 322540 | 324105 | trnP-a | 1566 |  | | | |
| 28 | 335257 | 338975 | trnH-cp | 3719 |  | | | |
| 29 | 373404 | 374739 | nad4-ex1 | 1336 |  | | | |
| 30 | 389952 | 391993 | trnfM, atp9 | 2042 |  | | | |
| 31 | 412028 | 413658 | trnK-cp intron | 1631 |  | | | |
| 32 | 418200 | 419095 | nad5 (last exon) | 896 |  | | | |
| 33 | 451320 | 457033 | repeat region (intergenic) | 5714 |  | | | |
| 34 | 456932 | 458609 | ccmC-nad1 spacer | 1678 |  | | | |
| 35 | 464238 | 467005 | nad1 intron | 2768 | % coverage | | | |
|  |  |  | Total= | 88653 | 18.91756361 | |  |  |

This table lists the start and end of all contiguous polyA read mapping sites with depth >500 reads along with any genes or notable featured within the mapping region.
