# Supplementary material for: The sugarcane mitochondrial genome: assembly, phylogenetics and transcriptomics
Source: PeerJ. 2019 Sep 24;7:e7558. doi: 10.7717/peerj.7558 (PMC6764373; doi:10.7717/peerj.7558)
Supplement: Supplemental Information 8 — Transposable elements in the sugarcane SP80-3280 mitochondrial genome. Transposable elements were derived from the Poaceae dataset of Censor. Column headings: Left and right, position of the transposable element in the mitochondrial genome (From/To indicates start/end of positions of the transposable elements). Orientation: + forward strand; −, complementary. "Sim" indicates value of similarity between two aligned fragments; "Pos" is the ratio of positives to alignment length; "Mm:Ts" is a ratio of mismatches to transitions in the nucleotide alignment. "Score," alignment score obtained from BLAST. [file peerj-07-7558-s008.docx]

Supplementary Table S4 — Transposable elements in the Sugarcane SP80-3280 mitochondrial genome

|  | Left Position | | Right Position | |  |  |  |  |  |  |
| --- | --- | --- | --- | --- | --- | --- | --- | --- | --- | --- |
| Chromosome  Name | From | To | From | To | Direction | Repbase library sequences | Class | Sim | Pos/Mm:Ts | Score |
| sp80-mt1 | 337 | 405 | 5666 | 5734 | + | MuDR-N206C_OS | DNA/MuDR | 0.971 | 2 | 581 |
| sp80-mt1 | 3123 | 3211 | 2438 | 2527 | - | MuDR-N206C_OS | DNA/MuDR | 0.9444 | 1 | 721 |
| sp80-mt1 | 5438 | 5566 | 3685 | 3818 | - | Copia-17_SB-I | LTR/Copia | 0.6718 | 1.8636 | 349 |
| sp80-mt1 | 8315 | 8368 | 5407 | 5460 | + | MuDR-N206C_OS | DNA/MuDR | 0.9815 | 1 | 470 |
| sp80-mt1 | 11506 | 11697 | 3791 | 3989 | - | INAV_HV_I | LTR/Copia | 0.6667 | 1.9355 | 459 |
| sp80-mt1 | 22769 | 23045 | 3997 | 4278 | + | MuDR-61_OS | DNA/MuDR | 0.9568 | 2.75 | 2330 |
| sp80-mt1 | 26207 | 26635 | 5 | 465 | - | Copia-33_BD-I | LTR/Copia | 0.7923 | 2.2903 | 1520 |
| sp80-mt1 | 26649 | 26705 | 8 | 77 | + | tRNA-Tyr_OS | Multicopy_gene/tRNA | 0.8103 | 1.25 | 263 |
| sp80-mt1 | 28527 | 28569 | 1504 | 1546 | + | Helitron-N136_OS | DNA/Helitron | 0.907 | 4 | 308 |
| sp80-mt1 | 30122 | 30168 | 1022 | 1070 | - | RLG_scDEL_1_6-LTR | LTR/Gypsy | 0.8367 | 6 | 264 |
| sp80-mt1 | 30353 | 30420 | 8 | 74 | + | tRNA-SerCGA_OS | Multicopy_gene/tRNA | 0.7941 | 1.5714 | 287 |
| sp80-mt1 | 30932 | 30992 | 2818 | 2877 | + | Copia-118_OS-I | LTR/Copia | 0.8361 | 1.5 | 357 |
| sp80-mt1 | 31411 | 31587 | 549 | 732 | + | RTE1_ZM | NonLTR/RTE | 0.7318 | 1.625 | 578 |
| sp80-mt1 | 33678 | 33724 | 2379 | 2425 | + | hAT-1_BDi | DNA/hAT | 0.7872 | 3.3333 | 238 |
| sp80-mt1 | 34186 | 34679 | 4152 | 4646 | + | ATLANTYS-I_OS | LTR/Gypsy | 0.6782 | 1.8571 | 1105 |
| sp80-mt1 | 35002 | 35067 | 2464 | 2529 | + | Copia-50_BD-I | LTR/Copia | 0.7727 | 1.5 | 338 |
| sp80-mt1 | 35487 | 35593 | 3384 | 3490 | + | LINE1-70_SBi | NonLTR/L1 | 0.7383 | 1.6471 | 470 |
| sp80-mt1 | 35598 | 35687 | 1455 | 1544 | + | EnSpm-41N_SBi | DNA/EnSpm/CACTA | 0.9778 | 1 | 823 |
| sp80-mt1 | 39379 | 39505 | 2162 | 2288 | + | MuDR-N206C_OS | DNA/MuDR | 0.8976 | 2.6 | 894 |
| sp80-mt1 | 50099 | 50169 | 2531 | 2601 | + | Gypsy-54_SB-I | LTR/Gypsy | 0.7778 | 3 | 243 |
| sp80-mt1 | 54170 | 54221 | 5459 | 5510 | + | MuDR-N206C_OS | DNA/MuDR | 0.9808 | 1 | 452 |
| sp80-mt1 | 62308 | 62342 | 4373 | 4409 | - | MuDR-N206C_OS | DNA/MuDR | 0.9444 | 1 | 271 |
| sp80-mt1 | 63629 | 63708 | 4269 | 4348 | + | MuDR-N206C_OS | DNA/MuDR | 0.975 | 2 | 686 |
| sp80-mt1 | 66445 | 66557 | 4014 | 4121 | - | Gypsy-188_OS-I | LTR/Gypsy | 0.7545 | 2 | 428 |
| sp80-mt1 | 68998 | 69039 | 4373 | 4414 | - | MuDR-N206C_OS | DNA/MuDR | 0.881 | 5 | 268 |
| sp80-mt1 | 69333 | 69359 | 1789 | 1815 | + | Helitron-1_BDi | DNA/Helitron | 0.963 | 1 | 241 |
| sp80-mt1 | 72935 | 72977 | 7017 | 7059 | + | MuDR-N206C_OS | DNA/MuDR | 0.9767 | 99 | 363 |
| sp80-mt1 | 73510 | 73542 | 1228 | 1260 | + | MuDR-N206C_OS | DNA/MuDR | 0.9394 | 2 | 257 |
| sp80-mt1 | 75499 | 75761 | 6725 | 6987 | + | Gypsy29-ZM_I | LTR/Gypsy | 0.659 | 1.8864 | 556 |
| sp80-mt1 | 77501 | 77583 | 2820 | 2900 | - | Gypsy-7_Sit-I | LTR/Gypsy | 0.7349 | 1.9 | 270 |
| sp80-mt1 | 85604 | 85656 | 9627 | 9679 | + | HELITRON2_OS | DNA/Helitron | 0.7736 | 2.4 | 250 |
| sp80-mt1 | 88913 | 89081 | 2819 | 2990 | - | Copia-59_ZM-I | LTR/Copia | 0.703 | 1.75 | 385 |
| sp80-mt1 | 89423 | 89453 | 1063 | 1093 | + | MuDR-N284_OS | DNA/MuDR | 0.9032 | 1 | 225 |
| sp80-mt1 | 92204 | 92276 | 3851 | 3923 | - | MuDR-N206C_OS | DNA/MuDR | 0.9726 | 2 | 619 |
| sp80-mt1 | 97562 | 97587 | 5064 | 5089 | - | MuDR-N206C_OS | DNA/MuDR | 1 | 99 | 234 |
| sp80-mt1 | 97691 | 97716 | 5064 | 5089 | - | MuDR-N206C_OS | DNA/MuDR | 1 | 99 | 234 |
| sp80-mt1 | 97865 | 97917 | 3994 | 4046 | + | MuDR-N206C_OS | DNA/MuDR | 0.9811 | 1 | 461 |
| sp80-mt1 | 98857 | 99065 | 4563 | 4769 | - | Copia-18_BD-I | LTR/Copia | 0.7464 | 1.7 | 862 |
| sp80-mt1 | 100394 | 100433 | 5719 | 5757 | - | MuDR-N206C_OS | DNA/MuDR | 0.95 | 99 | 299 |
| sp80-mt1 | 109433 | 109498 | 3135 | 3200 | - | MuDR-N206C_OS | DNA/MuDR | 0.7576 | 4 | 265 |
| sp80-mt1 | 114765 | 114805 | 1504 | 1544 | - | Helitron-N136_OS | DNA/Helitron | 0.9512 | 99 | 331 |
| sp80-mt1 | 118972 | 119051 | 860 | 935 | - | MERMITE18F | DNA/MuDR | 0.7179 | 1.6667 | 247 |
| sp80-mt1 | 123318 | 123386 | 1987 | 2055 | + | MuDR-N206C_OS | DNA/MuDR | 1 | 99 | 621 |
| sp80-mt1 | 125564 | 125649 | 4017 | 4100 | + | Copia-71_SB-I | LTR/Copia | 0.7241 | 1.5833 | 266 |
| sp80-mt1 | 126119 | 126193 | 581 | 658 | - | EnSpm-N1_AT | DNA/EnSpm/CACTA | 0.7105 | 1.6667 | 206 |
| sp80-mt1 | 132362 | 132453 | 2405 | 2494 | + | Helitron-N124B_OS | DNA/Helitron | 0.9121 | 2.3333 | 641 |
| sp80-mt1 | 132454 | 133063 | 4608 | 5217 | + | MuDR-64_OS | DNA/MuDR | 0.9738 | 1.3333 | 5458 |
| sp80-mt1 | 134689 | 134878 | 9221 | 9401 | - | Helitron-1_BDi | DNA/Helitron | 0.9185 | 2.4 | 1385 |
| sp80-mt1 | 137494 | 137561 | 2634 | 2695 | - | Helitron-N134_OS | DNA/Helitron | 0.7812 | 1.5714 | 256 |
| sp80-mt1 | 138622 | 138706 | 4857 | 4932 | - | MuDR-N206C_OS | DNA/MuDR | 0.8846 | 1.75 | 470 |
| sp80-mt1 | 139271 | 139370 | 291 | 387 | + | Helitron-N8B_OS | DNA/Helitron | 0.7396 | 2 | 214 |
| sp80-mt1 | 141588 | 141636 | 2620 | 2663 | + | LINE1-5_ZM | NonLTR/L1 | 0.8444 | 1.5 | 253 |
| sp80-mt1 | 144664 | 144745 | 6040 | 6116 | - | DANIELA_TM_I | LTR/Gypsy | 0.7308 | 1.8182 | 278 |
| sp80-mt1 | 145781 | 145908 | 4277 | 4404 | - | MuDR-61_OS | DNA/MuDR | 0.9922 | 1 | 1190 |
| sp80-mt1 | 146840 | 146909 | 833 | 902 | + | MuDR-N206C_OS | DNA/MuDR | 0.9571 | 1.5 | 580 |
| sp80-mt1 | 147337 | 147400 | 1918 | 1981 | + | MuDR-N206C_OS | DNA/MuDR | 0.9688 | 99 | 529 |
| sp80-mt1 | 151600 | 151657 | 5033 | 5090 | + | MuDR-N206C_OS | DNA/MuDR | 0.8103 | 2.75 | 306 |
| sp80-mt1 | 154448 | 154562 | 6561 | 6669 | + | EnSpm-3_HV | DNA/EnSpm/CACTA | 0.7297 | 1.7143 | 301 |
| sp80-mt1 | 154591 | 154783 | 2486 | 2700 | - | Copia-1_TA-I | LTR/Copia | 0.7041 | 2.0417 | 392 |
| sp80-mt1 | 155633 | 155726 | 9309 | 9401 | - | Helitron-1_BDi | DNA/Helitron | 0.9149 | 3.5 | 716 |
| sp80-mt1 | 157311 | 157404 | 691 | 790 | - | LINE1-9_ZM | NonLTR/L1 | 0.7083 | 1.5333 | 277 |
| sp80-mt1 | 157438 | 157551 | 802 | 924 | - | LINE1-35_ZM | NonLTR/L1 | 0.7105 | 2 | 317 |
| sp80-mt1 | 160388 | 160462 | 2971 | 3050 | - | MuDR-N206C_OS | DNA/MuDR | 0.9605 | 2 | 591 |
| sp80-mt1 | 164738 | 164855 | 2719 | 2830 | - | Copia-118_OS-I | LTR/Copia | 0.9646 | 1 | 916 |
| sp80-mt1 | 165411 | 165459 | 3806 | 3854 | - | MuDR-N206C_OS | DNA/MuDR | 0.9184 | 2 | 365 |
| sp80-mt1 | 166841 | 166919 | 4330 | 4409 | + | MuDR-N206C_OS | DNA/MuDR | 0.875 | 2 | 503 |
| sp80-mt1 | 169655 | 169738 | 3540 | 3623 | - | MuDR-N206C_OS | DNA/MuDR | 0.9286 | 2 | 648 |
| sp80-mt1 | 169916 | 169953 | 3092 | 3129 | + | Gypsy-13_Sit-I | LTR/Gypsy | 0.8684 | 1.6667 | 264 |
| sp80-mt1 | 174913 | 175064 | 2792 | 2944 | + | LINE1-26_SBi | NonLTR/L1 | 0.6883 | 1.875 | 423 |
| sp80-mt1 | 175558 | 175593 | 6149 | 6184 | - | MuDR-N206C_OS | DNA/MuDR | 1 | 99 | 324 |
| sp80-mt1 | 184345 | 184433 | 2194 | 2275 | + | EnSpm-1_HV | DNA/EnSpm/CACTA | 0.7262 | 1.5833 | 254 |
| sp80-mt1 | 198862 | 199459 | 3742 | 4344 | - | rn_179-105_IR | LTR/Copia | 0.7496 | 2.0286 | 2402 |
| sp80-mt1 | 199560 | 199631 | 3206 | 3278 | + | MuDR-N206C_OS | DNA/MuDR | 0.863 | 2.25 | 440 |
| sp80-mt1 | 200705 | 200795 | 918 | 1016 | - | hAT-12_SBi | DNA/hAT | 0.7312 | 1.9091 | 270 |
| sp80-mt1 | 201763 | 201803 | 2310 | 2350 | + | Copia-1_BDi-I | LTR/Copia | 0.8049 | 1.3333 | 235 |
| sp80-mt1 | 205814 | 205885 | 3499 | 3565 | + | EnSpm-14_ZM | DNA/EnSpm/CACTA | 0.7536 | 1.6667 | 261 |
| sp80-mt1 | 208757 | 208858 | 1045 | 1146 | + | MuDR-N206C_OS | DNA/MuDR | 0.9902 | 1 | 902 |
| sp80-mt1 | 209625 | 209665 | 2838 | 2878 | + | Helitron-N132_OS | DNA/Helitron | 0.8293 | 1.1667 | 240 |
| sp80-mt1 | 210350 | 210488 | 1463 | 1600 | + | MuDR-N206C_OS | DNA/MuDR | 0.9928 | 99 | 1214 |
| sp80-mt1 | 211746 | 211867 | 4325 | 4446 | - | L1-6_ZM | NonLTR/L1 | 0.712 | 2 | 271 |
| sp80-mt1 | 213370 | 213409 | 681 | 715 | + | MuDR-N265_OS | DNA/MuDR | 0.9444 | 99 | 237 |
| sp80-mt1 | 216716 | 216780 | 5074 | 5133 | + | BAGY2_HV_I | LTR/Gypsy | 0.7419 | 1.1667 | 237 |
| sp80-mt1 | 219334 | 219425 | 1798 | 1890 | + | Helitron-N138_OS | DNA/Helitron | 0.7766 | 1.5 | 422 |
| sp80-mt1 | 226124 | 226192 | 5560 | 5631 | - | LINE1-5_SBi | NonLTR/L1 | 0.7324 | 1.4545 | 264 |
| sp80-mt1 | 230753 | 230811 | 250 | 308 | - | MuDR-N1_ZM | DNA/MuDR | 0.7627 | 1.2727 | 314 |
| sp80-mt1 | 236986 | 237031 | 5335 | 5382 | - | Gypsy-95_ZM-I | LTR/Gypsy | 0.8085 | 2 | 225 |
| sp80-mt1 | 237389 | 237546 | 584 | 746 | + | SC-10_I | LTR/Copia | 0.7862 | 1.55 | 703 |
| sp80-mt1 | 239267 | 239301 | 4373 | 4409 | - | MuDR-N206C_OS | DNA/MuDR | 0.9444 | 1 | 271 |
| sp80-mt1 | 241599 | 241689 | 4697 | 4787 | - | MuDR-N206C_OS | DNA/MuDR | 0.967 | 1.5 | 763 |
| sp80-mt1 | 241915 | 241954 | 5795 | 5834 | + | MuDR-N206C_OS | DNA/MuDR | 0.925 | 1.5 | 308 |
| sp80-mt1 | 250965 | 250999 | 4373 | 4409 | - | MuDR-N206C_OS | DNA/MuDR | 0.9444 | 1 | 271 |
| sp80-mt1 | 251970 | 252062 | 1845 | 1942 | + | TOS17 | LTR/Copia | 0.7629 | 2 | 325 |
| sp80-mt1 | 255000 | 255118 | 43 | 152 | - | Helitron-N12_OS | DNA/Helitron | 0.7143 | 1.4444 | 283 |
| sp80-mt1 | 256534 | 256601 | 4877 | 4945 | - | LINE1-6_OS | NonLTR/L1 | 0.7681 | 3 | 277 |
| sp80-mt1 | 262979 | 263028 | 2832 | 2881 | - | Copia-118_OS-I | LTR/Copia | 0.96 | 99 | 403 |
| sp80-mt1 | 263515 | 263599 | 5093 | 5183 | + | MuDR-N206C_OS | DNA/MuDR | 0.908 | 2 | 588 |
| sp80-mt1 | 272627 | 272755 | 2248 | 2372 | + | Helitron-N67_OS | DNA/Helitron | 0.8672 | 1.3 | 751 |
| sp80-mt1 | 274709 | 274768 | 4 | 63 | + | tRNA-Asn_OS | Multicopy_gene/tRNA | 0.7742 | 1.25 | 228 |
| sp80-mt1 | 275415 | 275483 | 8 | 75 | - | tRNA-Thr_OS | Multicopy_gene/tRNA | 0.7857 | 2.2 | 276 |
| sp80-mt1 | 276119 | 276457 | 3087 | 3429 | - | Copia-91_SB-I | LTR/Copia | 0.6968 | 1.9583 | 874 |
| sp80-mt1 | 278024 | 278089 | 2700 | 2765 | + | Gypsy-24_BD-I | LTR/Gypsy | 0.7576 | 2.2857 | 277 |
| sp80-mt1 | 278932 | 279069 | 756 | 893 | - | MuDR-N265_OS | DNA/MuDR | 0.971 | 1.3333 | 1153 |
| sp80-mt1 | 279243 | 279272 | 4375 | 4404 | - | MuDR-N206C_OS | DNA/MuDR | 1 | 99 | 270 |
| sp80-mt1 | 279302 | 279379 | 2568 | 2645 | - | Gypsy67-ZM_I | LTR/Gypsy | 0.7179 | 1.4667 | 337 |
| sp80-mt1 | 280087 | 280140 | 903 | 956 | - | Copia-34_SB-I | LTR/Copia | 0.7593 | 1.625 | 246 |
| sp80-mt1 | 280505 | 280589 | 6238 | 6322 | + | MuDR-N206C_OS | DNA/MuDR | 0.9647 | 1.5 | 710 |
| sp80-mt1 | 283361 | 283422 | 2607 | 2669 | + | RETROSAT-3B_I | LTR/Gypsy | 0.7619 | 1.4 | 284 |
| sp80-mt1 | 285060 | 285132 | 1721 | 1793 | - | Gypsy-145_OS-I | LTR/Gypsy | 0.7397 | 1.9 | 305 |
| sp80-mt1 | 285323 | 285357 | 4373 | 4409 | - | MuDR-N206C_OS | DNA/MuDR | 0.9444 | 1 | 271 |
| sp80-mt1 | 285448 | 285473 | 5064 | 5089 | - | MuDR-N206C_OS | DNA/MuDR | 1 | 99 | 234 |
| sp80-mt1 | 285623 | 285675 | 3994 | 4046 | + | MuDR-N206C_OS | DNA/MuDR | 0.9811 | 1 | 461 |
| sp80-mt1 | 286615 | 286823 | 4563 | 4769 | - | Copia-18_BD-I | LTR/Copia | 0.7464 | 1.7 | 862 |
| sp80-mt1 | 288152 | 288191 | 5719 | 5757 | - | MuDR-N206C_OS | DNA/MuDR | 0.95 | 99 | 299 |
| sp80-mt1 | 297191 | 297256 | 3135 | 3200 | - | MuDR-N206C_OS | DNA/MuDR | 0.7576 | 4 | 265 |
