# Supplementary figures and images for: The sugarcane mitochondrial genome: assembly, phylogenetics and transcriptomics
Source: PeerJ. 2019 Sep 24;7:e7558. doi: 10.7717/peerj.7558 (PMC6764373; doi:10.7717/peerj.7558)

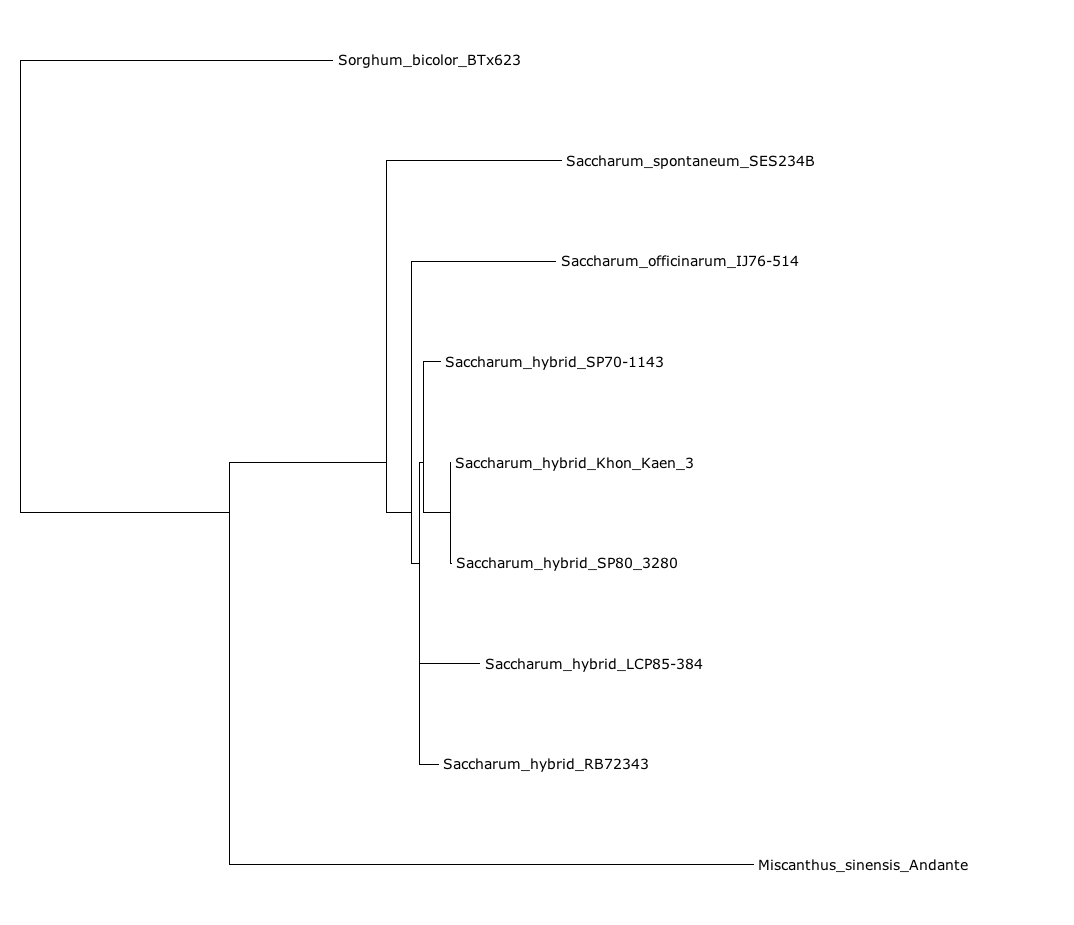

Supplement: Supplemental Information 9 — Tarball of all the sequence data from this project to be submitted or having been submitted but not yet available to ENA. The main directory contains two sub-directories for Dryad submissions and ENA submissions. All directories contain a readme file describing the contents. These are all the sequences and alignments from this study that will be made available through third party repositories (Dryad and ENA). [file peerj-07-7558-s009.gz › reviewer_supplementary_files/Dryad/Phylos/mt2/mt2-rooted-ML.png]

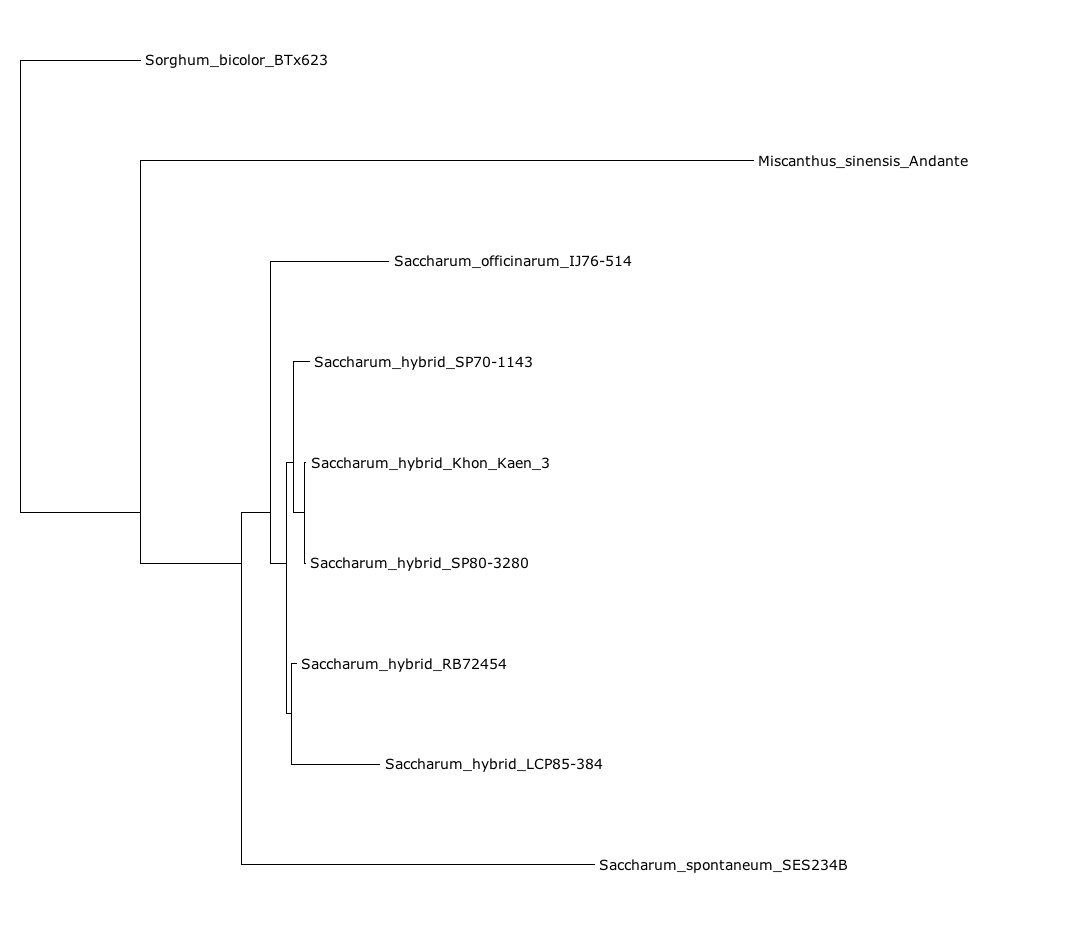

Supplement: Supplemental Information 9 — Tarball of all the sequence data from this project to be submitted or having been submitted but not yet available to ENA. The main directory contains two sub-directories for Dryad submissions and ENA submissions. All directories contain a readme file describing the contents. These are all the sequences and alignments from this study that will be made available through third party repositories (Dryad and ENA). [file peerj-07-7558-s009.gz › reviewer_supplementary_files/Dryad/Phylos/mt1/mt1-final-ML.png]

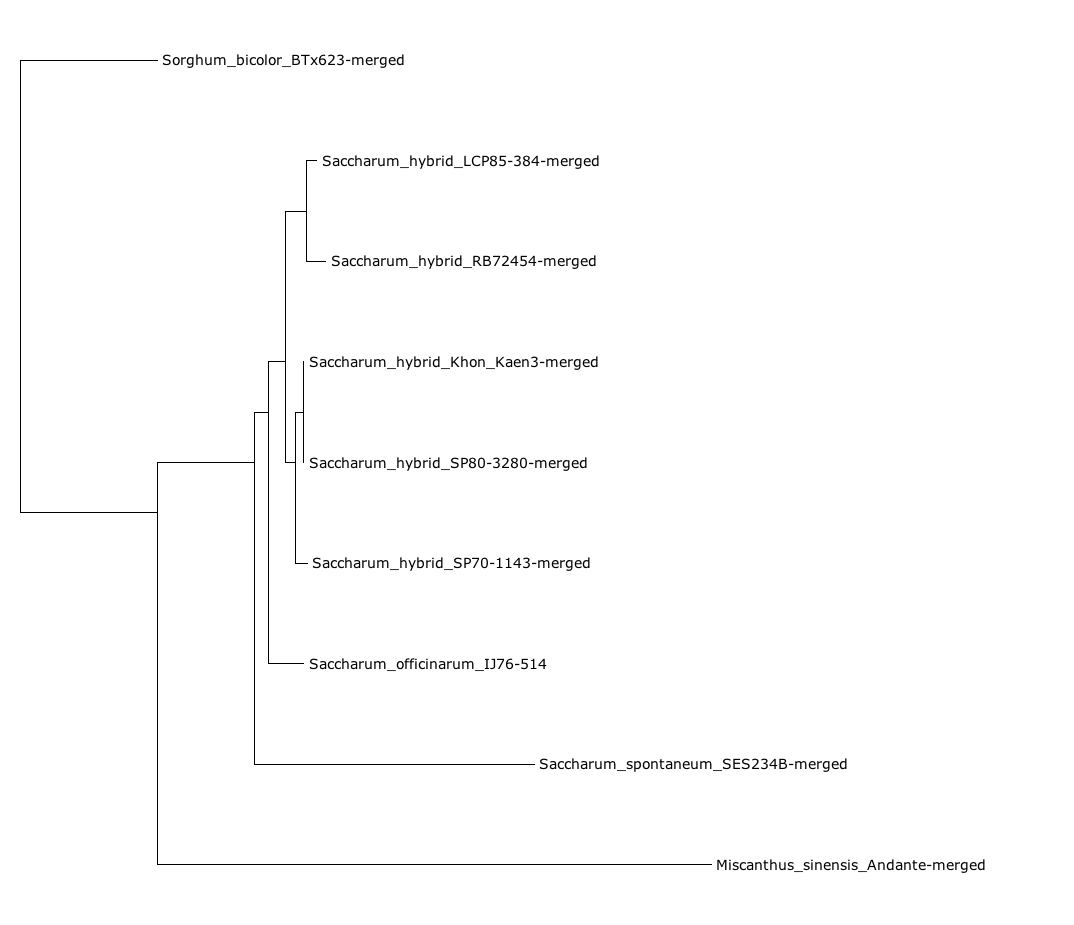

Supplement: Supplemental Information 9 — Tarball of all the sequence data from this project to be submitted or having been submitted but not yet available to ENA. The main directory contains two sub-directories for Dryad submissions and ENA submissions. All directories contain a readme file describing the contents. These are all the sequences and alignments from this study that will be made available through third party repositories (Dryad and ENA). [file peerj-07-7558-s009.gz › reviewer_supplementary_files/Dryad/Phylos/merged/MTs_Merged-ML.png]
